# Human liver sinusoidal endothelial cells promote intracellular crawling of lymphocytes during recruitment: A new step in migration

**DOI:** 10.1002/hep.28879

**Published:** 2016-11-25

**Authors:** Daniel A. Patten, Garrick K. Wilson, Dalan Bailey, Robert K. Shaw, Sirpa Jalkanen, Marko Salmi, Antal Rot, Chris J. Weston, David H. Adams, Shishir Shetty

**Affiliations:** ^1^ National Institute for Health Research Birmingham Liver Biomedical Research Unit and Centre for Liver Research, Medical School University of Birmingham Birmingham United Kingdom; ^2^ National Heart and Lung Institute, Imperial Centre for Translational and Experimental Medicine Imperial College London London United Kingdom; ^3^ Institute of Immunology and Immunotherapy, Institute of Biomedical Research University of Birmingham Birmingham United Kingdom; ^4^ Technology Hub Imaging Facility, Infrastructure and Facilities, College of Medical and Dental Sciences University of Birmingham Birmingham United Kingdom; ^5^ MediCity Research Laboratory, and Department of Medical Microbiology and Immunology University of Turku Turku Finland; ^6^ Centre for Immunology and Infection, Department of Biology University of York York United Kingdom

## Abstract

The recruitment of lymphocytes via the hepatic sinusoidal channels and positioning within liver tissue is a critical event in the development and persistence of chronic inflammatory liver diseases. The hepatic sinusoid is a unique vascular bed lined by hepatic sinusoidal endothelial cells (HSECs), a functionally and phenotypically distinct subpopulation of endothelial cells. Using flow‐based adhesion assays to study the migration of lymphocytes across primary human HSECs, we found that lymphocytes enter into HSECs, confirmed by electron microscopy demonstrating clear intracellular localization of lymphocytes *in vitro* and by studies in human liver tissues. Stimulation by interferon‐γ increased intracellular localization of lymphocytes within HSECs. Furthermore, using confocal imaging and time‐lapse recordings, we demonstrated “intracellular crawling” of lymphocytes entering into one endothelial cell from another. This required the expression of intracellular adhesion molecule‐1 and stabilin‐1 and was facilitated by the junctional complexes between HSECs. *Conclusion:* Lymphocyte migration is facilitated by the unique structure of HSECs. Intracellular crawling may contribute to optimal lymphocyte positioning in liver tissue during chronic hepatitis. (Hepatology 2017;65:294‐309).

**Abbreviations**
CLEVER‐1: common lymphathic endothelial and vascular endothelial receptor‐1
HSEC: human sinusoidal endothelial cell
HUVEC: human umbilical vein endothelial cells
ICAM‐1: intracellular adhesion molecule‐1
IFNγ: interferon‐γ
JAM‐A: junctional adhesion molecule‐A
PDL1: programmed death ligand‐1
TNFα: tumor necrosis factor α
ZO‐1: zona occludens‐1

Chronic inflammation is a major cause of global morbidity and mortality, often leading to tissue fibrosis and organ failure, and is also a recognized risk factor for carcinogenesis.^1^, ^2^, ^3^, ^4^ It is characterized by the recruitment of immune cells into organs via their interaction with endothelial cells followed by their positioning in strategic locations within the tissue.^5^ This is seen in nearly all adult liver diseases that are driven by chronic inflammation, where leukocytes are recruited via specialized channels known as sinusoids, which are lined by hepatic sinusoidal endothelial cells (HSECs).^6^ This influx of immune cells often leads to lymphoid aggregates/follicles around the portal tract in a range of liver diseases.^7^

Despite this common pathway of chronic inflammation, several features contribute to the liver being a unique site for leukocyte recruitment. The extravasation occurs within the hepatic sinusoidal channels in contrast to the postcapillary venules as seen in most other organs.^8^, ^9^ These channels are characterized by a low flow environment, and the sinusoidal endothelium (i.e., HSECs) has a unique morphology and performs specialized functions including scavenging and filtration.^10^ Conventional adhesion molecules, such as selectins which mediate leukocyte rolling, are absent from this vascular bed, and recruitment is mediated by atypical adhesion molecules such as vascular adhesion protein‐1 and the common lymphatic endothelial and vascular endothelial receptor‐1 (CLEVER‐1) also known as stabilin‐1.^11^, ^12^

The aim of this study was to perform a detailed analysis of the transendothelial pathway used by lymphocytes to cross human liver sinusoidal endothelium to identify organ‐specific targets of chronic inflammation within the liver. We developed real‐time cell imaging by laser scanning confocal microscopy under conditions of physiologically relevant shear stress to visualize the migration of lymphocytes across HSECs. We visualized lymphocyte migration into the cytoplasm of HSECs from where the cells crossed junctional membranes to allow them to crawl from within one HSEC into another. We noted this process more frequently in HSECs compared with conventional vascular endothelium and found it was enhanced by interferon‐γ (IFNγ) treatment of the endothelium. Although crawling of leukocytes on the luminal surface has been described previously,^13^ we believe this is the first description of “intracellular crawling,” which may play an important role in leukocyte recruitment and positioning within the liver.

## Materials and Methods

### HUMAN TISSUE

Human tissue and blood samples were collected from patients admitted to the University Hospitals Birmingham National Health Service Foundation Trust. Liver tissue was taken from organ donors that was surplus for surgical requirements or from uninvolved liver removed at hepatic resection for secondary liver tumors; diseased tissue was obtained from patients undergoing liver transplantation for chronic liver disease. Tissue samples from patients were obtained with written informed consent and with local ethics committee approval (reference numbers 06/Q2702/61 and 04/Q2708/41, South Birmingham, Birmingham, UK).

#### ENDOTHELIAL CELL ISOLATION

HSECs were isolated from approximately 30 g human liver tissue as described previously.^14^ Briefly, tissue was subjected to collagenase digestion (10 mg/mL collagenase IA; Sigma‐Aldrich, Gillingham, Dorset, UK) and was placed on a 33%/77% Percoll (Amersham Biosciences, Little Chalfont, Buckinghamsire, UK) density gradient. The nonparenchymal cell layer was then removed, and the endothelial cells were isolated by positive immunomagnetic selection utilizing CD31 antibody‐conjugated Dynabeads (Thermo Fisher, Bishop Meadow Road, Loughborough, UK). The endothelial cells were then cultured in medium composed of human endothelial basal growth medium (Thermo Fisher) supplemented with 10% human serum (HD Supplies, Botolph Claydon, Buckinghamshire, UK), 10 ng/mL vascular endothelial growth factor (PeproTech, London, UK), and 10 ng/mL hepatocyte growth factor (PeproTech). The cells were grown in rat tail culture vessels coated with collagen (1 in 100; Sigma‐Aldrich) and were maintained at 37°C in a humidified incubator with 5% CO_2_. Human umbilical vein endothelial cells (HUVECs) isolated using standard methods^14^ were used as a control endothelial cell line.

### FLOW‐BASED ADHESION ASSAY

To study lymphocyte migration in the adhesion cascade within the hepatic sinusoids, cytokine‐stimulated HSECs (tumor necrosis factor α [TNFα] and IFNγ for 24 hours at 10 ng/mL) were grown to confluence in μ‐slide VI chambers (ibidi, Thistle Scientific, Uddingston, Glasgow, UK) and connected to the flow system described previously.^15^ In some experiments, IFNγ stimulation was performed for shorter periods (2 and 4 hours). For live cell imaging of lymphocyte migration we used a slightly modified flow‐assay protocol to that used previously. Cytokine‐stimulated HSECs or HUVECs were seeded into μ‐slide I chambers (ibidi) and were prelabeled with CellTracker Green CMFDA (Thermo Fisher). The chambers were then connected to an ibidi pump, which allowed continuous perfusion of lymphocytes at a shear stress of 0.05Pa. Flow assays were performed with lymphocytes prelabeled with CellTracker Violet BMQC (Thermo Fisher) according to the manufacturer's guidelines. In some assays, the endothelial cells were labeled with a Cell Mask plasma membrane stain according to the manufacturer's guidelines. Some flow assays were performed with nonviable lymphocytes (cells underwent fixation with 4% paraformaldehyde). The endothelial monolayers and adherent lymphocytes were visualized and examined using a Zeiss 780 Zen microscope equipped with a 63 × 1.32 objective. Time‐lapsed confocal images and z‐stacks were acquired and analyzed using Zen software.

#### STATISTICAL ANALYSIS

All data are expressed as the mean ± SEM for the specified number of experimental repeats (*n*). For single comparisons, statistical significance was determined using an unpaired *t* test, whereas evaluation of multiple treatments was performed using analysis of variance with a Tukey's *post hoc* multiple comparison test. A *P* value of ≤ 0.05 was considered statistically significant. All statistical analyses were performed using Prism 6 software (GraphPad Software Inc.) group.

Further details are provided in the http://onlinelibrary.wiley.com/doi/10.1002/hep.28879/suppinfo.

## Results

### INTRAENDOTHELIAL LYMPHOCYTES IN CHRONIC INFLAMMATORY LIVER DISEASE

**Figure 1:**
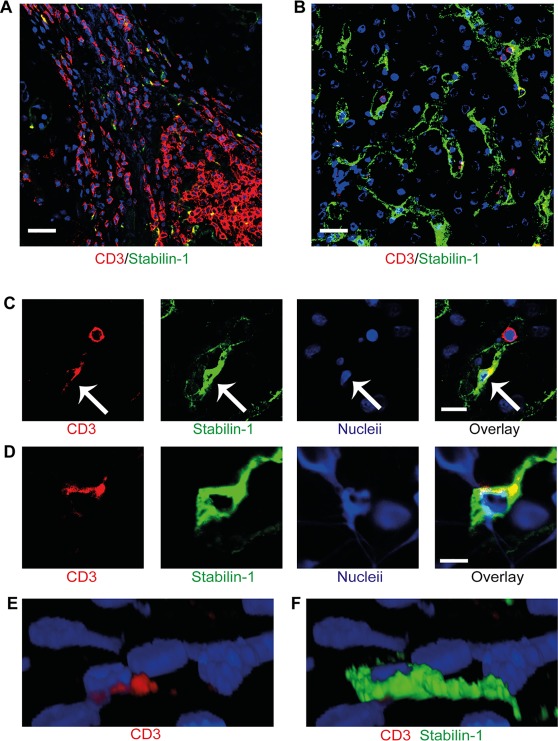
Lymphocytes migrate into sinusoidal endothelial cells in human chronic liver disease. (A) Immunofluorescent staining of liver sections from a patient with primary biliary cirrhosis demonstrating the presence of lymphocytes in portal regions within fibrous septum. (B) Immunofluorescent staining of the same liver demonstrating lymphocytes within the sinusoidal channels. CD3+ lymphocytes appear in red and stabilin‐1–positive hepatic sinusoidal endothelium appears in green. (C) Lymphocytes (red) within the sinusoidal channels with one lymphocyte within the sinusoidal lumen and another within the endothelial cell (green). Arrows indicate the intraendothelial lymphocyte. (D) Two‐dimensional image of orthogonal (XZ) projection of a CD3+ lymphocyte (red) colocalizing with a stabilin‐1–positive (green) endothelial cell. (E,F) Three‐dimensional reconstruction of the orthogonal (XZ) projection in panel D with CD3+ red signal only (E) and overlay of stabilin‐1–positive green signal (F). Scale bars = 20 μm (A,B), 10 μm (C), and 5 μm (D).

Many chronic liver conditions are characterized by a portal lymphocyte infiltrate in close relation to the site of hepatic scar formation (Fig. 1A). Lymphocyte recruitment is initiated within the hepatic sinusoids,^8^ which are lined by specialized endothelia characterized by a unique phenotypic expression, including liver/lymph node–specific intercellular adhesion molecule‐3‐grabbing integrin and the scavenger receptor stabilin‐1 (Fig. 1B).^16^ Using flow adhesion assays with primary HSECs, we recently reported that a proportion of lymphocytes use a transcellular route to cross sinusoidal endothelium.^12^ To assess in human tissue, sections of livers from patients with chronic liver disease were visualized by confocal microscopy and immunofluorescent labeling. We used stabilin‐1 as a marker of sinusoidal endothelial cells and CD45 and CD3 to define lymphocytes, which allowed us to identify lymphocytes within the hepatic sinusoids and capture their interactions with lining endothelial cells (http://onlinelibrary.wiley.com/doi/10.1002/hep.28879/suppinfo). We visualized lymphocytes within the hepatic sinusoids and adherent to the endothelium; in addition, we identified lymphocytes that appeared to have migrated into the endothelial cytoplasm (Fig. 1C). Multilayer imaging of these CD3^+^ cells allowed three‐dimensional reconstruction to confirm that these cells were within sinusoidal endothelial cells (Fig. 1D‐F). We ruled out phagocytosis of lymphocytes by tissue resident macrophages by using the Kupffer cell marker CD68 to distinguish Kupffer cells from stabilin‐1–positive endothelial cells (http://onlinelibrary.wiley.com/doi/10.1002/hep.28879/suppinfo). These results demonstrate that the migration of lymphocytes into HSEC occurs *in situ* during chronic liver disease.

### IFNγ ACTIVATION OF LIVER SINUSOIDAL ENDOTHELIAL CELLS PROMOTES INTRACELLULAR MIGRATION OF LYMPHOCYTES

Having visualized the presence of lymphocytes within HSEC in human tissue we proceeded to study migration of lymphocytes across endothelial monolayers in flow based adhesion assays to study the molecular basis of lymphocyte recruitment under levels of physiological shear stress.^14^ In these assays HSEC require preactivation with cytokine to promote adhesion of lymphocytes. TNFα and IFNγ are known to be significantly up‐regulated in inflammatory liver disease and important activators of endothelium during cellular injury.^11^, ^17^

**Figure 2:**
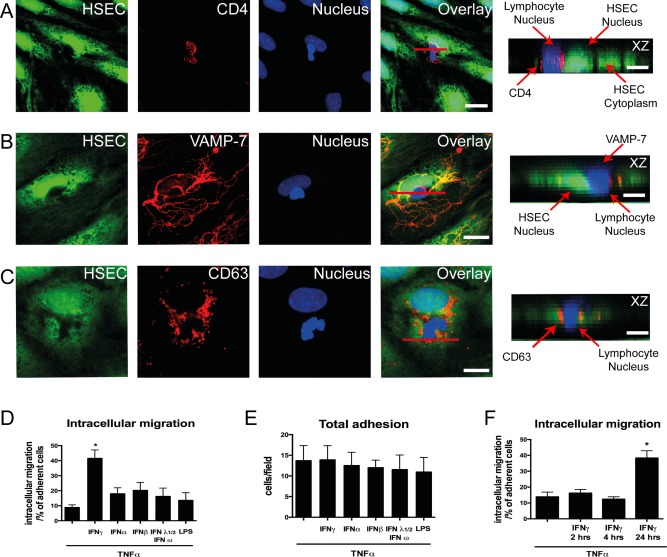
Intracellular migration of lymphocytes into primary HSECs. (A) Representative confocal images of lymphocytes adherent to cytokine‐treated HSEC monolayer. HSEC cytoplasm was stained with CellTracker CMFDA (green), lymphocyte membrane was stained with CD4 marker (red), and HSEC and lymphocyte nuclei were stained with DAPI (blue). (B) Immunofluorescent staining for lysosomal markers VAMP‐7 (red) and (C) CD63 (red) were performed. Orthogonal (XZ) projections are shown corresponding to the plane of the red line in the overlay images. Arrows in the orthogonal projections indicate the HSEC/lymphocyte nucleus and its relationship to the lysosomal compartment. (D) Quantification of intracellular migration. (E) Adhesion of peripheral blood lymphocytes on cytokine‐treated HSECs. (F) Quantification of intracellular migration on TNFα‐ and IFNγ‐treated HSECs at various time points. Quantitative data are the mean ± SEM of three independent experiments. Statistical significance was determined using one‐way analysis of variance, with a Tukey's *post hoc* multiple comparison test. *P < 0.05. Scale bars = 20 μm (A), 10 μm (B,C), and 5 μm (A‐C, orthogonal projections).

More recently, we have incorporated a “fixed cell” technique, where monolayers of HSECs were perfused with lymphocytes under shear stress underwent fixation and were stained with cellular dyes and specific antibodies, followed by analysis with confocal microscopy to study the route taken by lymphocytes during transendothelial migration across HSECs. Using this technique, incorporating cytoplasmic dyes and membrane markers such as CD4 to distinguish between HSECs and lymphocytes, we observed lymphocytes migrating directly into HSECs by way of the transcellular route (Fig. 2A). To confirm that migrating lymphocytes were within endothelial cell bodies, we stained intracellular structures focusing on the lysosomal compartment which is highly enriched in HSECs. By staining lysosomes with VAMP‐7 and CD63 we demonstrated that lymphocytes displace the lysosomal compartment during intracellular migration (Fig. 2B,C).

**Figure 3:**
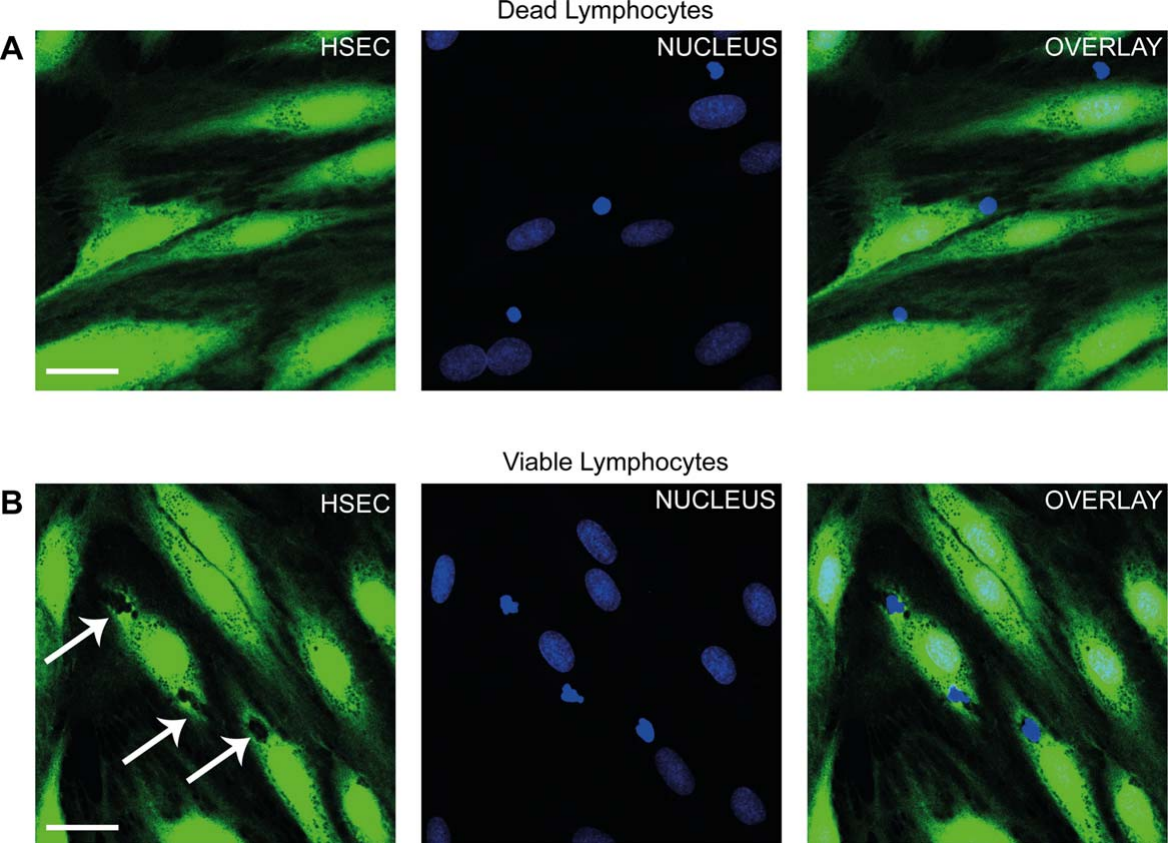
Nonviable lymphocytes are not internalized by HSECs. (A) Representative confocal images of nonviable (fixed) lymphocytes perfused over TNFα‐ and IFNγ‐treated HSECs in a flow adhesion assay. Endothelial cells were stained with CellTracker CFMDA (green) and nuclei were stained with DAPI (blue). (B) Repeat experiments with viable lymphocytes. Arrows indicate intracellular lymphocytes. Images are representative of three independent experiments. Scale bar = 30 μm (A,B).

**Figure 4:**
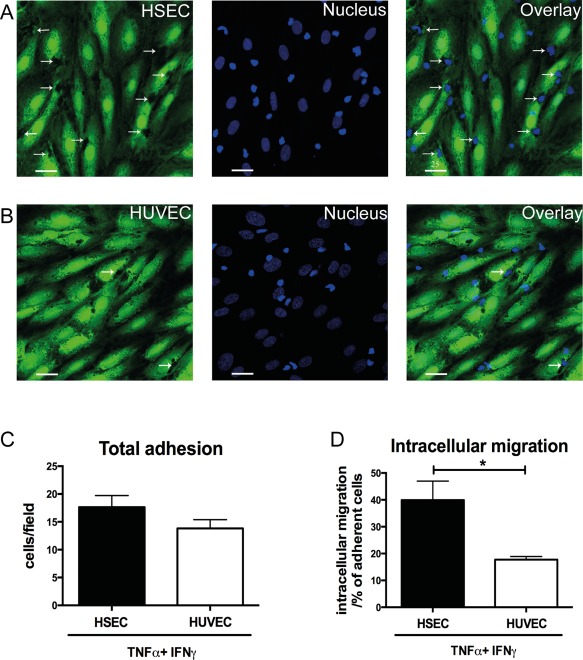
IFNγ promotes intracellular migration of lymphocytes into HSECs but not HUVECs. (A,B) Representative confocal images of lymphocytes adherent to TNFα‐ and IFNγ‐treated HSEC and HUVEC monolayers in a flow adhesion assay. Endothelial cells were stained with CellTracker CFMDA (green) and nuclei were stained with DAPI (blue). Arrows indicate intracellular lymphocytes. (C) Quantification of adhesion and (D) intracellular migration of lymphocytes into HSEC and HUVEC monolayers in a flow adhesion assay. Quantitative data are the mean ± SEM of five independent experiments. Statistical significance was determined using a two‐tailed *t* test. *P < 0.05. Scale bars = 25 μm (A,B).

We then quantified this route of migration by counting the proportion of total adherent cells captured migrating into HSECs at a specific time point. Very few lymphocytes were detected within HSECs after TNFα stimulation, but a significant number of cells were seen to use this route following the addition of IFNγ (Fig. 2D). Treatment of HSECs with other interferon family cytokines or lipopolysaccharide did not promote intracellular migration (Fig. 2D) despite leading to an increase in the number of adherent lymphocytes per field of similar magnitude to that seen with IFNγ (Fig. 2E). These experiments were performed after 24 hours of stimulation. To assess whether duration of IFNγ stimulation impacted on this migration experiments were done at various time points (2, 4, and 24 hours). We found that 24‐hour stimulation of IFNγ was required before significant numbers of lymphocytes underwent intracellular migration (Fig. 2F). To differentiate this process from phagocytosis, we performed our flow assays with nonviable (fixed) lymphocytes. We did not visualize any internalization of nonviable lymphocytes by HSECs, repeat experiments with viable lymphocytes demonstrated clear displacement of the HSEC cytoplasm (Fig. 3A,B). To determine whether this was a specific feature of HSECs, we tested the ability of IFNγ to promote this route of migration in vascular endothelial cells isolated from HUVECs. We found that whereas total adhesion was similar between HSECs and HUVECs, intracellular migration in HUVECs occurred significantly less frequently compared with HSECs (Fig. 4A‐D). These results demonstrate that IFNγ has a particular effect on lymphocyte migration across HSECs, which occurs less frequently during interactions with conventional vascular endothelium.

**Figure 5:**
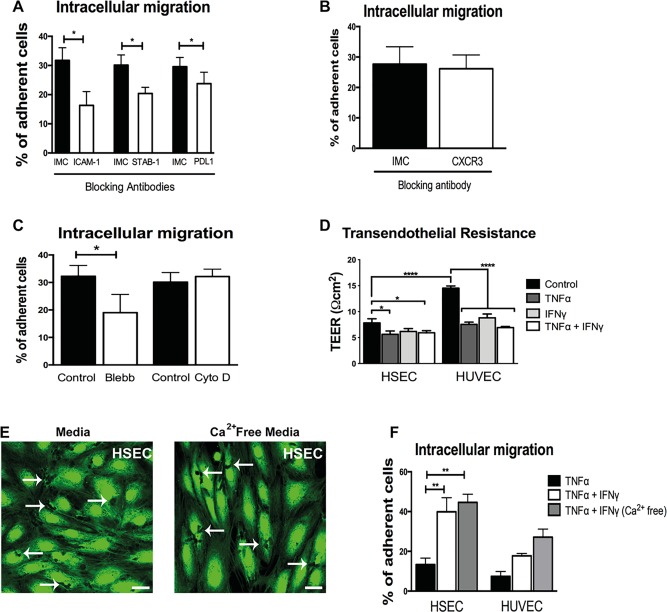
Intracellular migration is dependent on ICAM‐1 but not inhibited by disruption of junctional complexes. (A) Quantification of intracellular migration of lymphocytes across HSECs pretreated with blocking antibodies to ICAM‐1, CLEVER‐1/stabilin‐1 (STAB‐1), and PDL1. Results are the mean ± SEM of at least three independent experiments. (B) Quantification of intracellular migration of lymphocytes pretreated with CXCR3 blocking antibody or IMC across HSECs. Results are the mean ± SEM of at least three independent experiments. (C) Quantification of intracellular migration of lymphocytes across HSECs pretreated with control media or media supplemented with Blebbistatin (Blebb) or cytochalasin D (Cyto D). Results are the mean ± SEM of three independent experiments. (D) Measurement of transelectrical endothelial resistance across untreated or cytokine‐treated HSECs and HUVECs. Results are the mean ± SEM of six independent experiments. (E) Representative images of cell tracker CFMDA‐labeled endothelial monolayers (green) after flow assay pretreated with TNFα and IFNγ and cultured in flow media or calcium‐free flow media. Arrows indicate lymphocyte intracellular migration. (F) Quantification of intracellular migration of lymphocytes across cytokine‐treated HSEC and HUVEC monolayers cultured in flow media (Media) or calcium‐free flow media. Results are the mean ± SEM of three independent experiments. Statistical significance was determined using a two‐tailed *t* test (A‐C) and one‐way analysis of variance with a Tukey's *post hoc* multiple comparison test (D,F). Scale bar = 25 μm (E). *P < 0.05. **P < 0.005. ****P < 0.0005.

We assessed the contribution of adhesion molecules focusing on the receptors intracellular adhesion molecule‐1 (ICAM‐1) and stabilin‐1, both of which are involved in diapedesis of lymphocytes across HSECs,^12^ and programmed death ligand‐1 (PDL1), which is up‐regulated by IFNγ and contributes to leukocyte recruitment.^18^, ^19^ We found that blocking of ICAM‐1 or CLEVER‐1/stabilin‐1 led to a significant reduction of lymphocyte intracellular migration into HSECs and that blocking PDL1 had a small but significant effect (Fig. 5A). To study the role of interferon‐inducible chemokines (CXCL9‐11), we inhibited their receptor, CXCR3. Blocking CXCR3 in lymphocytes did not affect their ability to migrate into HSECs (Fig. 5B). Additionally, we measured transcription levels of CXCL9‐11 in IFNγ‐treated HUVECs compared with HSECs and observed significantly lower levels in HSECs compared with HUVECs (http://onlinelibrary.wiley.com/doi/10.1002/hep.28879/suppinfo). We proceeded to assess whether interferon‐inducible factors could have an indirect effect on the adhesion molecules that mediate intracellular migration, particularly ICAM‐1 and CLEVER‐1/stabilin‐1. Transcription levels of ICAM‐1 were significantly up‐regulated with TNFα, but IFNγ did not have an additive effect (http://onlinelibrary.wiley.com/doi/10.1002/hep.28879/suppinfo). Levels of CLEVER‐1/stabilin‐1 demonstrated a nonsignificant decreased trend in transcription with cytokine stimulation (http://onlinelibrary.wiley.com/doi/10.1002/hep.28879/suppinfo).

Intracellular migration is likely to require changes or reorganization in the cytoskeleton to allow migration within the endothelial cell. We initially assessed how cytokine stimulation affected key components of the cytoskeleton within HSECs. Imaging and quantification of actin and microtubule fibers demonstrated a significant reduction and reorganization of the cytoskeleton with TNFα (http://onlinelibrary.wiley.com/doi/10.1002/hep.28879/suppinfo). Further reduction of actin fibers was noted with the addition of IFNγ (http://onlinelibrary.wiley.com/doi/10.1002/hep.28879/suppinfo). We went on to study these cytoskeleton components during lymphocyte interaction with HSECs under shear stress. We did not detect lymphocytes interacting with endothelial actin during intracellular migration (http://onlinelibrary.wiley.com/doi/10.1002/hep.28879/suppinfo), but endothelial microtubule structures were enriched around lymphocytes, especially near endothelial junctions (http://onlinelibrary.wiley.com/doi/10.1002/hep.28879/suppinfo). We then studied the functional contribution of actin and microtubule formation to intracellular migration using cytochalasin D and Blebbistatin to inhibit actin polymerization and myosin II, respectively. Whereas pretreatment of endothelial cells with cytochalasin D had no impact, pretreatment with Blebbistatin significantly reduced intracellular migration (Fig. 5C). Collectively, these results demonstrate that lymphocyte migration into HSECs is mediated by a combination of typical and atypical adhesion molecules facilitated by cytoskeletal changes affecting predominantly the microtubule compartment.

To determine whether alterations in endothelial permeability explain the difference between HSECs and HUVECs, we used transendothelial electrical resistance to compare the permeability of HSEC and HUVEC monolayers. Although HSECs demonstrated a lower electrical resistance compared with HUVECs in the resting state, both endothelial types had similar permeability quantified by electrical resistance in response to IFNγ and TNFα stimulation (Fig. 5D). In addition, permeability measured using a fluorescein isothiocyanate–dextran assay was comparable between HUVECs and HSECs (http://onlinelibrary.wiley.com/doi/10.1002/hep.28879/suppinfo).

Changes in endothelial junctions are likely to be required for cell‐to‐cell migration, which led us to study endothelial junctional integrity in this process. We disrupted junctional complexes by preculturing the endothelial monolayers in calcium‐free media,^20^ but this had no effect on total number of lymphocytes adhering to the endothelial surface (http://onlinelibrary.wiley.com/doi/10.1002/hep.28879/suppinfo) or on the number of cells undergoing intracellular migration into HSECs (Fig. 5E,F). Our findings demonstrate that lack of junctional integrity does not prevent lymphocyte intracellular migration and that this migratory pathway appears to be independent of endothelial permeability.

### REAL‐TIME IMAGING UNDER CONDITIONS OF FLOW DEMONSTRATES A NOVEL STEP OF INTRACELLULAR LYMPHOCYTE CRAWLING

To study lymphocyte migration in real time, we developed a confocal microscopy technique to visualize lymphocyte/endothelial interactions under conditions of physiological shear. Time‐lapse recordings allowed the analysis of lymphocyte migration across HSECs in a selected field of view. These recordings confirmed our earlier findings with the fixed cell technique that treatment with TNFα led to minimal disruption of the endothelial cytoplasm during lymphocyte migration (http://onlinelibrary.wiley.com/doi/10.1002/hep.28879/suppinfo). In contrast, treatment with a combination of TNFα and IFNγ promoted intracellular migration of lymphocytes into HSECs and led to clear disruption of the cytoplasm (http://onlinelibrary.wiley.com/doi/10.1002/hep.28879/suppinfo).

**Figure 6:**
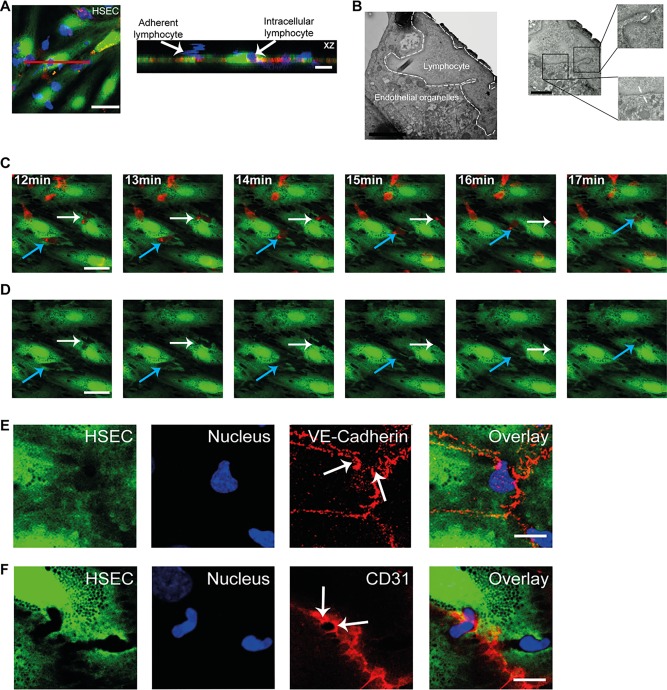
Intracellular crawling of lymphocytes across HSEC monolayers. (A) Live cell imaging of peripheral blood lymphocytes migrating across TNFα‐ and IFNγ‐treated HSECs under shear stress. HSECs were stained with CellTracker CFMDA (green), lymphocytes with cell tracker BMQC (blue), and HSEC junctions with CellMask orange plasma membrane stain (red). Orthogonal (XZ) projection is shown corresponding to the plane of the red line. (B) Left: Representative transmission electron microscopy image of an intracellular lymphocyte within TNFα‐ and IFNγ‐treated HSECs. Right: Arrows in high magnification images of two regions within the intracellular lymphocyte indicate a double membrane. (C) Still images of http://onlinelibrary.wiley.com/doi/10.1002/hep.28879/suppinfo taken at 1‐minute intervals of time‐lapse recordings of lymphocytes migrating across TNFα‐ and IFNγ‐stimulated HSEC monolayer under shear stress. HSEC cytoplasm was prelabeled with CellTracker CFMDA (green) and lymphocytes were prelabeled with CellTracker BMQC (red). (D) The same sequence of images shown in panel C with the red (lymphocyte) signal omitted. The arrows indicate lymphocytes (red) undergoing intracellular crawling from one endothelial cell to the adjacent cell displacing the cytoplasm of the endothelial cell (green). Note the redistribution of endothelial cytoplasm in panel D as the lymphocytes migrate from cell to cell. (E) Representative confocal images of lymphocyte cell‐to‐cell crawling across TNFα‐ and IFNγ‐treated HSEC monolayer. HSEC cytoplasm was stained with CellTracker CFMDA (green) and HSECs and lymphocyte nuclei were stained with DAPI (blue) and VE‐cadherin (red). Arrows indicate disruption of VE‐cadherin at the endothelial junction as lymphocytes migrate to the adjacent HSECs. (F) Representative confocal images of experiment with same conditions as panel D with staining of CD31 (red). Arrows indicate enrichment of CD31 at the junction as lymphocyte migrates to the adjacent HSECs. Scale bars = 25 μm (A,C,D), 10 μm (A, orthogonal projection, E,F), 2 μm (B, left), and 1 μm (B, right).

Thus far, our imaging of intracellular lymphocytes was on fixed samples; we then confirmed that we could visualize intracellular lymphocytes in live cells using z‐stack imaging in real time. The imaging confirmed lymphocytes within endothelial cells and we were able to clearly distinguish them from lymphocytes adherent to the endothelial surface (Fig. 6A). Although confocal microscopy demonstrated lymphocytes within liver endothelial cells, it could not define the ultrastructural changes involved in this process, leading us to undertake transmission electron microscopy of monolayers of HSECs treated with TNFα and IFNγ. Endothelial cells respond to cytokine activation by an increase in biosynthetic organelles,^21^ and this was evident in the HSEC ultrastructure visualized by transmission electron microscopy (http://onlinelibrary.wiley.com/doi/10.1002/hep.28879/suppinfo). When cytokine‐treated HSECs were incubated with lymphocytes, we successfully identified lymphocytes within endothelial structures, which were distinguishable from other endothelial organelles (Fig. 6B). A clear “double” membrane was visible around the lymphocytes (Fig. 6B), suggesting that intracellular migration of lymphocytes into HSECs is facilitated by the formation of intracellular vesicles.

Real‐time imaging with confocal microscopy highlighted a migratory pattern that could not be visualized using phase contrast microscopy or our fixed cell technique. Analysis of time‐lapse recordings demonstrated that a proportion of lymphocytes were migrating intracellularly and then crawling from the cytoplasm of one endothelial cell to another before completing transendothelial migration (http://onlinelibrary.wiley.com/doi/10.1002/hep.28879/suppinfo and Fig. 6C,D). Three‐dimensional reconstruction of the z‐stack images demonstrated lymphocytes within the endothelial cytoplasmic compartment in contrast to those crawling on the surface (http://onlinelibrary.wiley.com/doi/10.1002/hep.28879/suppinfo). To our knowledge, lymphocyte crawling from one endothelial cell to another intracellularly has never been described. A lymphocyte that crawls intracellularly between cells has to cross junctional barriers. To study this process, we returned to our fixed cell technique and used VE‐cadherin and CD31 as endothelial junctional markers. We were able to demonstrate lymphocytes migrating from cell to cell and crossing cell junctions; the VE‐cadherin complexes were clearly disrupted as lymphocytes migrated across the junction (Fig. 6E). In contrast, CD31 could be seen enriching the junctional pore as the lymphocyte migrated from one endothelial cell to the next (Fig. 6F). This appeared similar to findings described by previous groups demonstrating how CD31 is targeted to segments of the membrane during leukocyte–endothelial interactions.^22^

The flow adhesion assay performed in a microslide with a monolayer of endothelial cells does not incorporate a chemokine gradient or a substantial subendothelial compartment, and these features might inhibit transendothelial migration and promote intracellular crawling. To model this, we performed lymphocyte migration assays on a layer of HSECs that was mounted on a collagen plug containing the chemokine CXCL10 to provide a gradient and a subendothelial compartment, which would recapitulate the situation *in vivo* during lymphocyte recruitment. We found that despite the presence of the gradient and a subendothelial space, we still detected intracellular lymphocytes and also identified lymphocytes crossing HSEC junctions (http://onlinelibrary.wiley.com/doi/10.1002/hep.28879/suppinfo). Collectively, these results demonstrate a new step in the adhesion cascade wherein lymphocytes crawl intracellularly between endothelial cells.

### HUMAN LIVER SINUSOIDAL ENDOTHELIAL JUNCTIONAL MOLECULAR EXPRESSION DIFFERS FROM HUVECs

**Figure 7:**
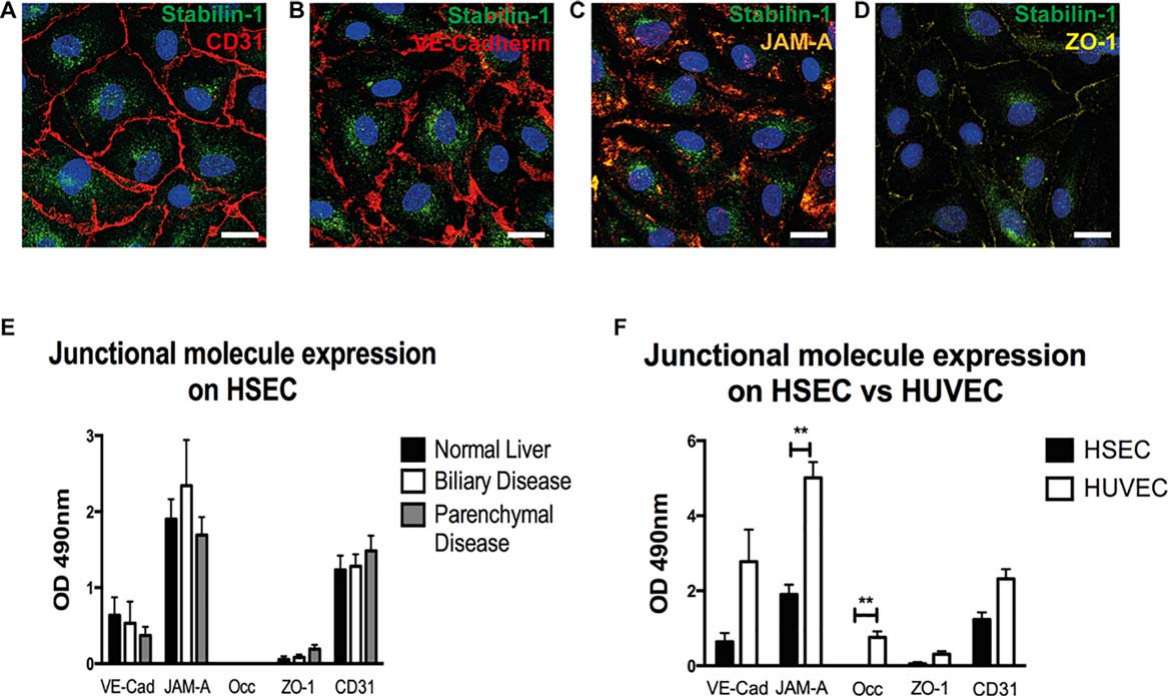
Junctional molecular expression differs between HSECs and HUVECs. (A‐D) Immunofluorescent staining of primary HSECs for CLEVER‐1/stabilin‐1 (green) and junctional molecules (red). Images representative of three separate HSEC isolates. (E) Cell‐based ELISA of junctional molecule expression in HSECs isolated from normal livers and chronically inflamed livers. (F) Cell‐based ELISA of junctional molecule expression in HSECs compared with HUVECs. Data are the mean of three experiments and values represent the mean optical density at 490 nm of three replicate wells minus the optical density of an isotype‐matched control antibody. Statistical significance was determined by two‐tailed *t* test. **P < 0.005. Scale bars = 20 μm (A‐D).

Our identification of intracellular cell‐to‐cell crawling associated with junctional disruption led us to compare in more detail the junctional complexes in HUVECs and HSECs. We obtained normal primary human HSECs from the margins of liver tissue resected to remove colorectal metastases and from unused donor organs. HSEC were also obtained from explanted liver tissue from patients with chronic liver disease. As we have shown previously,^12^ isolated HSECs express CD31 and the scavenger receptor stabilin‐1 (Fig. 7A). The junctional molecules identified in HSECs included VE‐cadherin, junctional adhesion molecule‐A (JAM‐A), and zona occludens‐1 (ZO‐1) (Fig. 7B‐D). Occludin, claudin‐1, and E cadherin were not detected. We next compared quantitative expression of junctional molecules in HSECs isolated from normal liver tissue, or tissue from patients with parenchymal liver disease (alcoholic liver disease and nonalcoholic fatty liver disease) or biliary disease (primary biliary cirrhosis, primary sclerosing cholangitis). We found that the profile and quantification of junctional molecular expression was very similar between the disease groups (Fig. 7E). We next assessed the effect of cytokine stimulation on junctional molecule expression. A combination of TNFα and IFNγ stimulation led to a small but nonsignificant reduction in junctional molecule expression (http://onlinelibrary.wiley.com/doi/10.1002/hep.28879/suppinfo). Finally, we made a direct comparison of junctional molecule expression between HSECs and HUVECs (Fig. 7F). We found quantitatively higher expression of JAM‐A in HUVECs compared with HSECs and occludin, an integral plasma membrane protein located at tight junctions, was found in HUVECs but was absent from HSECs. These studies confirm that the junctional molecule expression between HSECs from diseased and normal livers are similar in composition but are significantly different from HUVECs a prototypic venular endothelial cell.

### MICROARRAY ANALYSIS OF HSECs AND HUVECs CONFIRMS SIGNIFICANT TRANSCIPTOME DIFFERENCES IN MOLECULES INVOLVED IN JUNCTIONAL COMPLEX FORMATION AND REGULATION OF CELLULAR CYTOSKELETON

**Figure 8:**
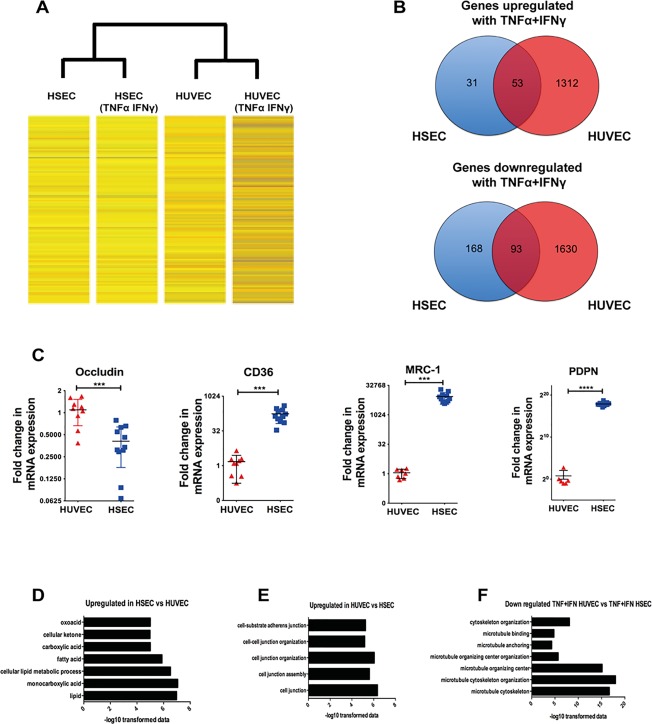
Microarray demonstrates differential gene expression changes between HSECs and HUVECs in response to TNFα and IFNγ challenge. (A) Heat map images of gene expression changes. Unstimulated HSECs and HUVECs were compared with HSECs and HUVECs that had been stimulated with TNFα and IFNγ (10 ng/mL) for 24 hours. (B) Summary of the total number of mutual and exclusively up‐regulated and down‐regulated genes after cytokine stimulation in HSECs and HUVECs. Genes were identified to be up‐regulated or down‐regulated based on log ratios that were 2‐fold or greater. (C) Comparative messenger RNA expression between HUVECs and HSECs of occludin, CD36, macrophage mannose receptor (MRC‐1), and podoplanin (PDPN). (D,E) Pathway analysis of up‐regulated and down‐regulated genes in unstimulated HSECs compared with HUVECs. (F) Pathway analysis of down‐regulated genes in stimulated HUVECs compared with stimulated HSECs. The pathways are plotted against their corresponding −log10 values of probability on the *x* axis. Statistical significance was determined using a two‐tailed *t* test. ***P < 0.001. ****P < 0.0005.

To further explore the potential mechanisms mediating intracellular crawling, we compared gene expression between HSECs and HUVECs using microarray analysis. Cytokine stimulation resulted in more genes being up‐regulated in HUVECs than in HSECs (Fig. 8A). Whereas over 1000 genes were up‐regulated in HUVECs, fewer genes were up‐regulated in HSECs in response to TNFα and IFNγ, and 31 were exclusive to HSECs (Fig. 8B). Pathway analysis did not identify an enriched pathway within this group of up‐regulated genes.

We then compared the transcription profile of unstimulated HSECs and HUVECs and found 1164 genes significantly up‐regulated in HSECs compared with HUVECs and 1346 genes up‐regulated in HUVECs compared with HSECs. We validated the microarray findings by confirming differences in messenger RNA between HSECs and HUVECs by way of quantitative polymerase chain reaction for four genes differentially expressed in the microarray: occludin, CD36, macrophage mannose receptor, and podoplanin (Fig. 8C). Down‐regulation of occludin at the transcript level further supported our earlier junctional protein comparison of HSECs with HUVECs (Fig. 7F).

Pathway analysis demonstrated that the majority of processes up‐regulated in HSECs were related to metabolism including lipids, fatty acids, carboxylic acid, ketones, and oxoacid metabolic processes (Fig. 8D and http://onlinelibrary.wiley.com/doi/10.1002/hep.28879/suppinfo). A total of 175 pathways were found to be significantly up‐regulated in HUVECs and included cell junction assembly, cell junction organization, and adherens junction organization (Fig. 8E and http://onlinelibrary.wiley.com/doi/10.1002/hep.28879/suppinfo). A total of 1630 genes were exclusively down‐regulated in HUVECs after cytokine stimulation, and a pathway enrichment analysis of this group of genes (http://onlinelibrary.wiley.com/doi/10.1002/hep.28879/suppinfo) revealed that several of these pathways were related to the cellular cytoskeleton, especially the microtubule compartment, including regulation of microtubule cytoskeleton organization, microtubule anchoring, microtubule polymerization, and microtubule organization (Fig. 8F). Collectively, these results support our findings that HUVECs and HSECs are characterized by significant differences in junctional formation.

## Discussion

The endothelium plays an active role in directing and selecting leukocyte subset recruitment to tissues from blood.^23^ A key step in this process is diapedesis, and the conventional pathway is the paracellular route where a leukocyte migrates between endothelial cells at cellular junctions.^24^ Although a transcellular route was described more than 40 years ago, it was only recently confirmed that leukocytes could migrate by this second route,^25^ and the reasons why leukocytes take a paracellular rather than a transcellular route are poorly understood.^26^ Endothelial heterogeneity is likely to contribute, because previous studies have demonstrated that transcellular migration occurs at a much higher frequency in endothelium lining microvascular beds,^25^ including our own work in HSECs.^12^ We now extend these findings to demonstrate a new migratory pattern of intracellular crawling of lymphocytes through the body of liver sinusoidal endothelial cells to adjacent endothelial cells, which is promoted by IFNγ treatment of endothelial cells. We have shown this using real‐time imaging of liver cells under conditions of physiological flow. Confocal microscopy and ultrastructural studies demonstrate that lymphocytes within endothelial cells are surrounded by a double membrane and displace endothelial organelles as they migrate. These intracellular lymphocytes migrate to other liver endothelial cells by disrupting the VE‐cadherin network, which is also implicated in paracellular leukocyte migration.^27^ This form of migration also involves the enrichment of CD31 at intercellular junctions and the contribution of the microtubule cytoskeleton. Changes in the endothelial cytoskeleton are presumably required to allow the lymphocytes to migrate through the cytoplasm. Migration via the lateral bordering recycling compartment, which has been shown to contribute to leukocyte paracellular and transcellular migration, is characterized by mobilization of CD31 and the microtubule network and our data suggest this compartment also regulates intracellular crawling.^28^ Our findings that intracellular crawling still occurred despite the blockade of CXCR3 suggests that this process is independent of the previously described pathway of lymphocyte migration mediated by intracellular chemokine stores,^29^ but we cannot rule out other indirect effects of interferon inducible factors on this step in migration. Our experiments demonstrated that IFNγ did not significantly increase the transcription of ICAM‐1 or CLEVER‐1/stabilin‐1, but we have previously shown that TNFα and IFNγ stimulation of HSECs can promote cell surface expression of CLEVER‐1/stabilin‐1.^12^ The effects of IFNγ on this step of migration required 24‐hour stimulation and were not seen with shorter times. Cytokine stimulation of endothelium can be characterized as type I or type II activation^30^ where type II activation occurs with longer periods of stimulation characterized by increased gene transcription and *de novo* protein synthesis, characterized by hypertrophy of the endothelial cell and increased biosynthetic organelle formation.^31^ Our findings suggest that type II activation is required for intracellular crawling.

The phenomenon was seen far less frequently in HUVECs, suggesting that the unique phenotype of HSECs, particularly differences in junctional complexes, plays a major role in this phenomenon. Liver sinusoidal endothelial cells have distinct molecular complexes at their junctions compared with vascular endothelium, showing much lower levels of VE‐cadherin, JAM‐A, ZO‐1, CD31, and absent occludin.^32^ These differences were maintained in HSECs isolated from patients with chronic inflammatory diseases. Conventional endothelial tight junctions are likely to be required for “directed” leukocyte migration through the paracellular route. This is supported by recent findings with a blood–brain barrier model characterized by robust tight junctions where >98% of transendothelial migration was paracellular.^33^ Additionally, our study shows that if membranes are disrupted by calcium‐free media, intracellular migration is increased. Thus, the relative lack of tight junction molecules in sinusoidal endothelium may actively promote both transcellular migration and intracellular crawling between endothelial cells.

Why should intracellular lymphocyte migration be a characteristic of hepatic sinusoidal endothelium? One explanation may be the important role played by sinusoidal endothelium in the liver in removing particulates and other factors that constantly enter the liver from the gut via the portal vein. Hepatic sinusoidal endothelium expresses a large number of scavenger receptors that are not found on vascular endothelium and has the capability to remove, internalize, and process or degrade a range of antigens. It also plays an important role in determining the nature of the adaptive immune response to such antigens by inducing antigen‐specific tolerance in T lymphocytes. It is thus possible that the intracellular migration we have observed contributes to this function by allowing lymphocytes to perform immune surveillance for intracellular pathogens.

Our findings may also demonstrate a key step in the lymphocyte migratory route through the liver. Lymphocytes continually recirculate through the liver under steady‐state conditions. Although hepatic sinusoids have been established as the major site of leukocyte recruitment, the migratory route taken by lymphocytes through the liver is poorly understood. Different zones within the hepatic acinus are associated with different hepatocyte functions defined by differential expression of, for instance, cytochrome P450 enzymes. Lymphocytes may thus need to be directed to particular compartments within the hepatic parenchyma to provide efficient detection and clearance of pathogens or harmful antigens. Furthermore, most forms of hepatitis are associated with a portal infiltrate even when the presumed antigen is confined to hepatocytes (e.g., viral hepatitis or autoimmune hepatitis). Kinetic studies in experimental models have demonstrated that lymphocytes migrate from the hepatic sinusoids to the portal tract and then drain to hepatic lymph nodes.^34^ During chronic inflammatory diseases lymphocyte recruitment increases often leading to portal infiltrates.^35^ However, the route taken by lymphocytes from the sinusoids to the portal tracts is not known. Migration from the hepatic sinusoids to the portal tract may occur via the space of Disse, the subendothelial layer containing the hepatic pericyte or stellate cell, and this appears to be the route taken by activated dendritic cells to exit the liver parenchyma and enter portal tracts. Our findings suggest that lymphocytes may also crawl intracellularly from endothelial cell to endothelial cell toward the portal regions. This route may become more relevant during chronic disease when collagen accumulates in the space of Disse, which may make it difficult for lymphocytes to use this pathway to portal tracts. The fact that we saw a marked increase in intravascular crawling in HSECs treated with a combination of TNFα and IFNγ supports a particular role in inflammation. We therefore believe this novel finding provides a new insight into lymphocyte migration and could lead to new treatments for inflammatory liver diseases.

## Supporting information

Additional Supporting Information may be found at http://onlinelibrary.wiley.com/doi/10.1002/hep.28879/suppinfo.

Supporting Information

Supporting Information

Supporting Information

Supporting Information

Supporting Information

Supporting Information
