# Supplementary material for: Human liver sinusoidal endothelial cells promote intracellular crawling of lymphocytes during recruitment: A new step in migration
Source: Hepatology. 2016 Nov 25;65(1):294–309. doi: 10.1002/hep.28879 (PMC5321563; doi:10.1002/hep.28879)
Supplement: Supplementary file 1 — Supporting Information [file HEP-65-294-s001.pdf]

Supplementary Movies, Figures and Tables

Movie 1

Time lapse recordings of lymphocyte migration across TNFα stimulated HSEC monolayer under shear stress. Lymphocytes were prelabelled with a CellTracker BMQC (blue) and HSEC prelabelled with CellTracker CFMDA (green). Lymphocytes (blue) can be seen crawling below and above the endothelial surface (green). The video is a representative recording from 3 separate experiments with different HSEC.

Movie 2

Time lapse recordings of endothelial monolayer during lymphocyte migration across TNFα stimulated HSEC under shear stress. Same sequence as in Movie 1 with the blue (lymphocyte) signal omitted. Note the endothelial cytoplasm is undisturbed during lymphocyte crawling and migration.

Movie 3

Time lapse recordings of lymphocyte migration across TNFα and IFNγ stimulated HSEC monolayer under shear stress. Lymphocytes were prelabelled with CellTracker BMQC (blue) and HSEC prelabelled with CellTracker CFMDA (green). Lymphocytes (blue) can be seen crawling below and above the endothelial surface (green) but also migrating intracellularly. The video is a representative recording from 3 separate experiments with different HSEC.

Movie 4

Time lapse recordings of endothelial monolayer during lymphocyte migration across TNFα and IFNγ stimulated HSEC under shear stress. Same sequence as in Movie 3 with the blue (lymphocyte) signal omitted. Note the redistribution of the endothelial cytoplasm (green) during lymphocyte crawling and migration.

Movie 5

Time lapse recordings of lymphocyte migration across TNFα and IFNγ stimulated HSEC monolayer under shear stress demonstrating 'intracellular crawling'. Lymphocytes were prelabelled with CellTracker BMQC (orange) and HSEC prelabelled with CellTracker CMFDA (green). White border box demonstrates lymphocytes redistributing endothelial cytoplasm during crawling from one to endothelial cell to another.

Supplementary Figure 1

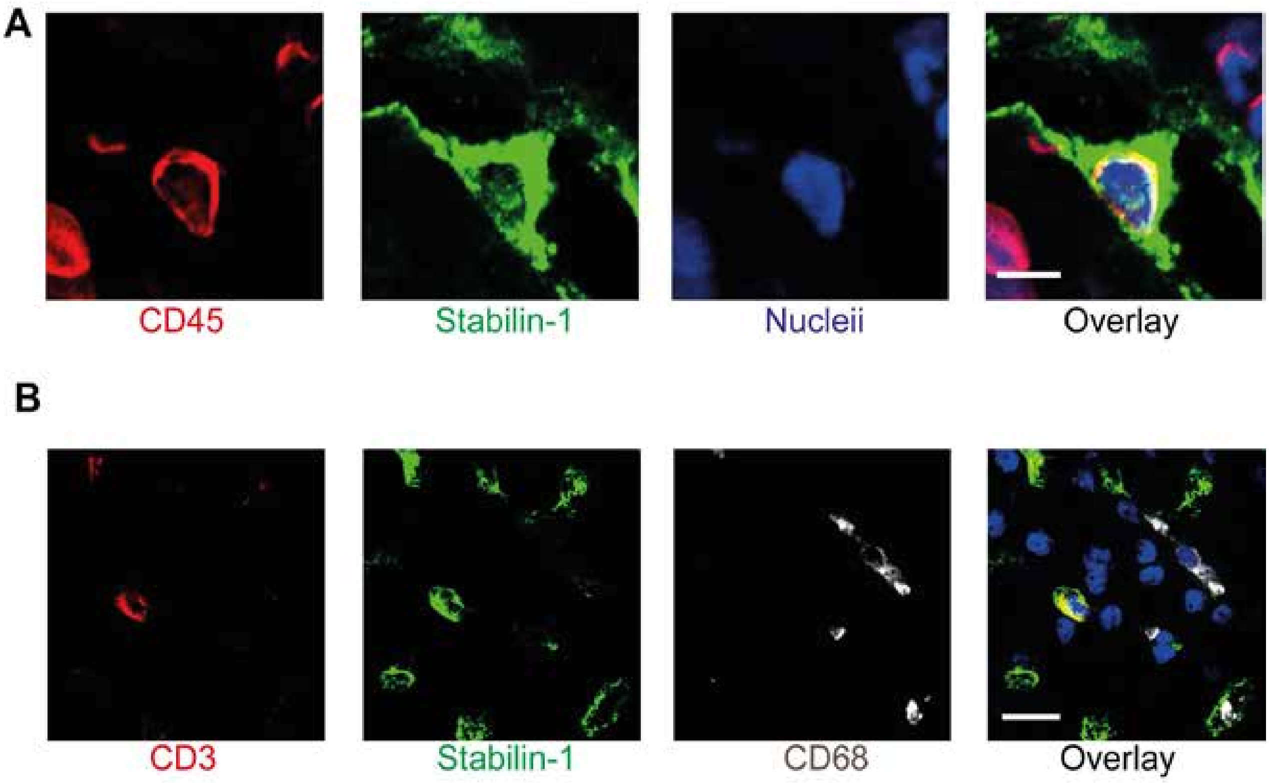

Stabilin-1 is restricted to endothelial cells within the hepatic sinusoids and not expressed on Kupffer cells. (A) High magnification confocal imaging of liver tissue with immunofluorescent staining demonstrating a $CD45^{+}$ (red) cell within a stabilin-1^{+} (green) endothelial cell. (B) Immunofluorescent imaging of human liver section distinguishing endothelial cells (green) and lymphocytes (red) from CD68+ Kupffer cells (grey). Images are representatives of three separate liver sections. Bar 5 µm (A), 10 µm (B).

Supplementary Figure 2

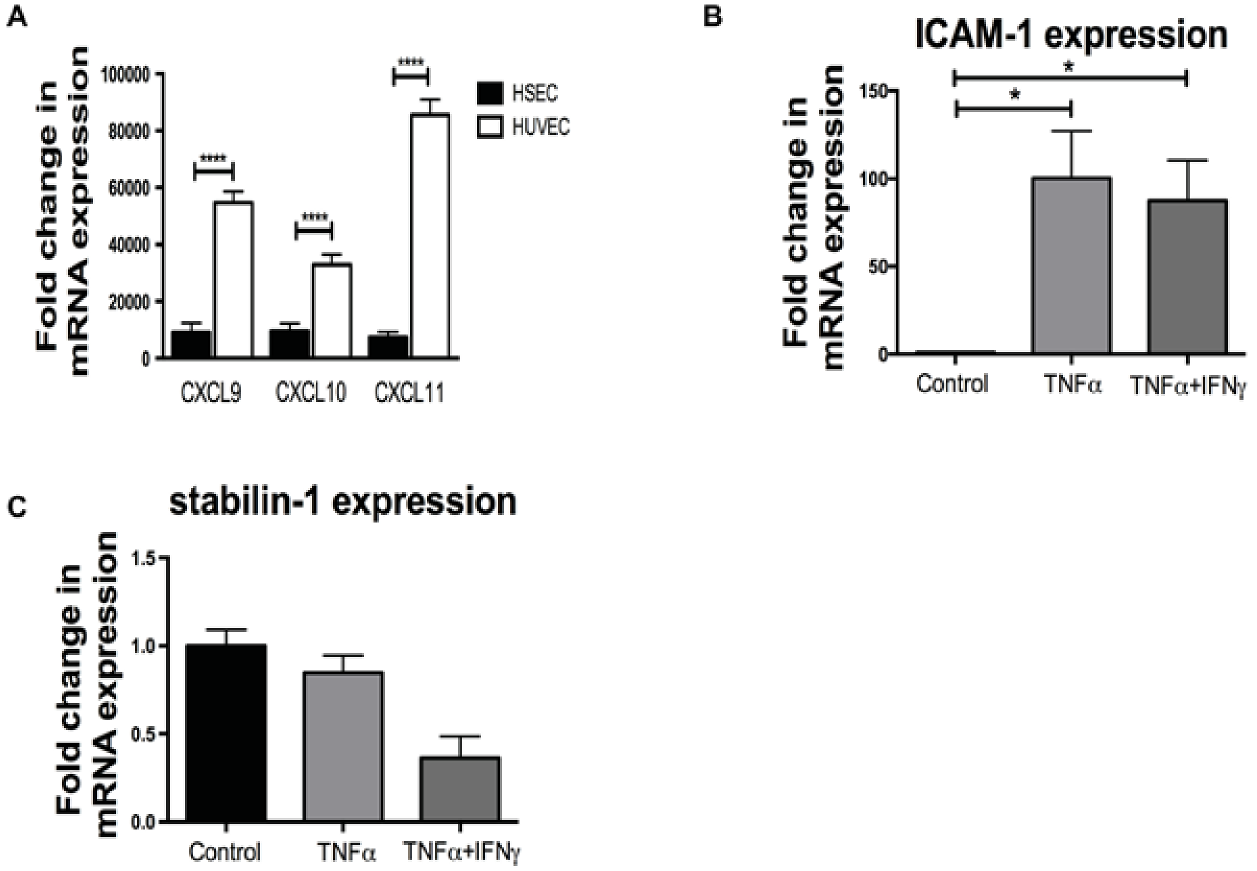

Chemokine expression and adhesion molecule expression in response to TNFα and IFNγ (A) mRNA expression of CXCL9-11 in TNFα and IFNγ treated HSEC and HUVEC, the results are normalized to untreated HSEC and are shown as the mean ± SEM of four independent experiments. (B) mRNA expression of ICAM-1 in TNFα and TNFα and IFNγ treated HSEC, the results are normalized to untreated HSEC (control) and are shown as the mean of ± SEM of five independent experiments. (C) mRNA expression of CLEVER-1/Stabilin-1 in TNFα and TNFα and IFNγ treated HSEC, results are normalized to untreated HSEC (control) and are shown as the mean of ± SEM of three independent experiments Statistical significance was determined by 1-way ANOVA analysis, with a Tukey's *post-hoc* multiple comparison test . * P<0.05, ****P<0.0005.

Supplementary Figure 3

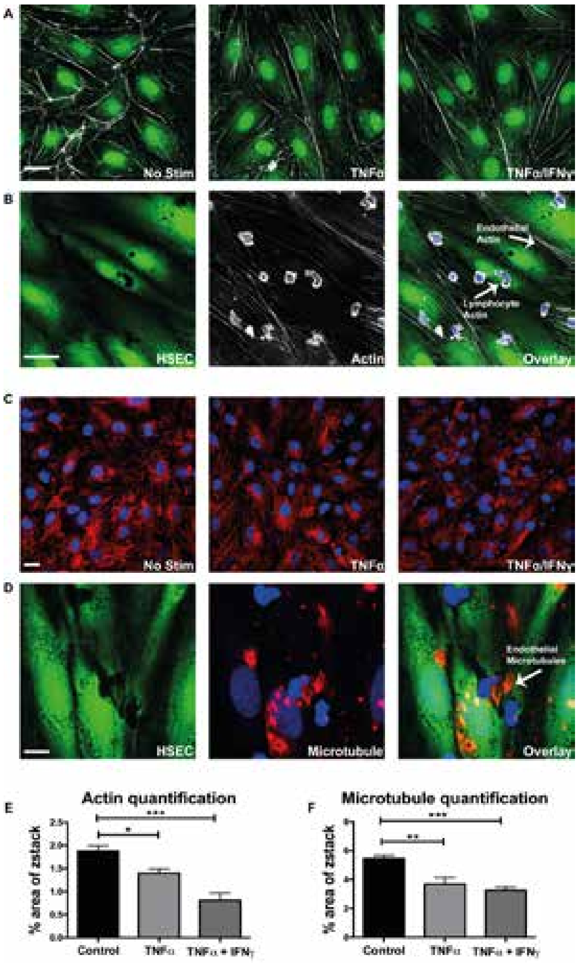

Actin and Microtubule expression in cytokine treated HSEC and during lymphocyte migration.

(A) Representative images of immunofluorescent staining of unstimulated and cytokine stimulated monolayers of HSEC with actin (grey) and HSEC prelabelled with Celltracker CFMDA (green). (B) Representative confocal images of lymphocytes adherent to TNFα and IFNγ treated HSEC monolayers after being perfused under shear stress. Endothelial cells were pre-labelled with CellTracker CFMDA (green), nuclei with DAPI (blue) and immunofluorescent staining with actin (grey). (C) Representative images of immunofluorescent staining of unstimulated and cytokine stimulated monolayers of HSEC with microtubules (red) and HSEC nuclei labeled with DAPI (blue). (D) Representative confocal images of lymphocytes adherent to TNFα and IFNγ treated HSEC monolayers after being perfused under shear stress. Endothelial cells were pre-labelled with CellTracker CFMDA (green), nuclei with DAPI (blue) and immunofluorescent staining of microtubules (red). (E,F) Quantification of actin and microtubule fibres in unstimulated and cytokine stimulated HSEC, results are the mean % area ± SEM of ten random orthogonal projections taken from five separate fields for each group. *$P<0.05$, ** $P<0.005$. Bar 20 μm (A,B,C) Bar 10 μm.

Supplementary Figure 4

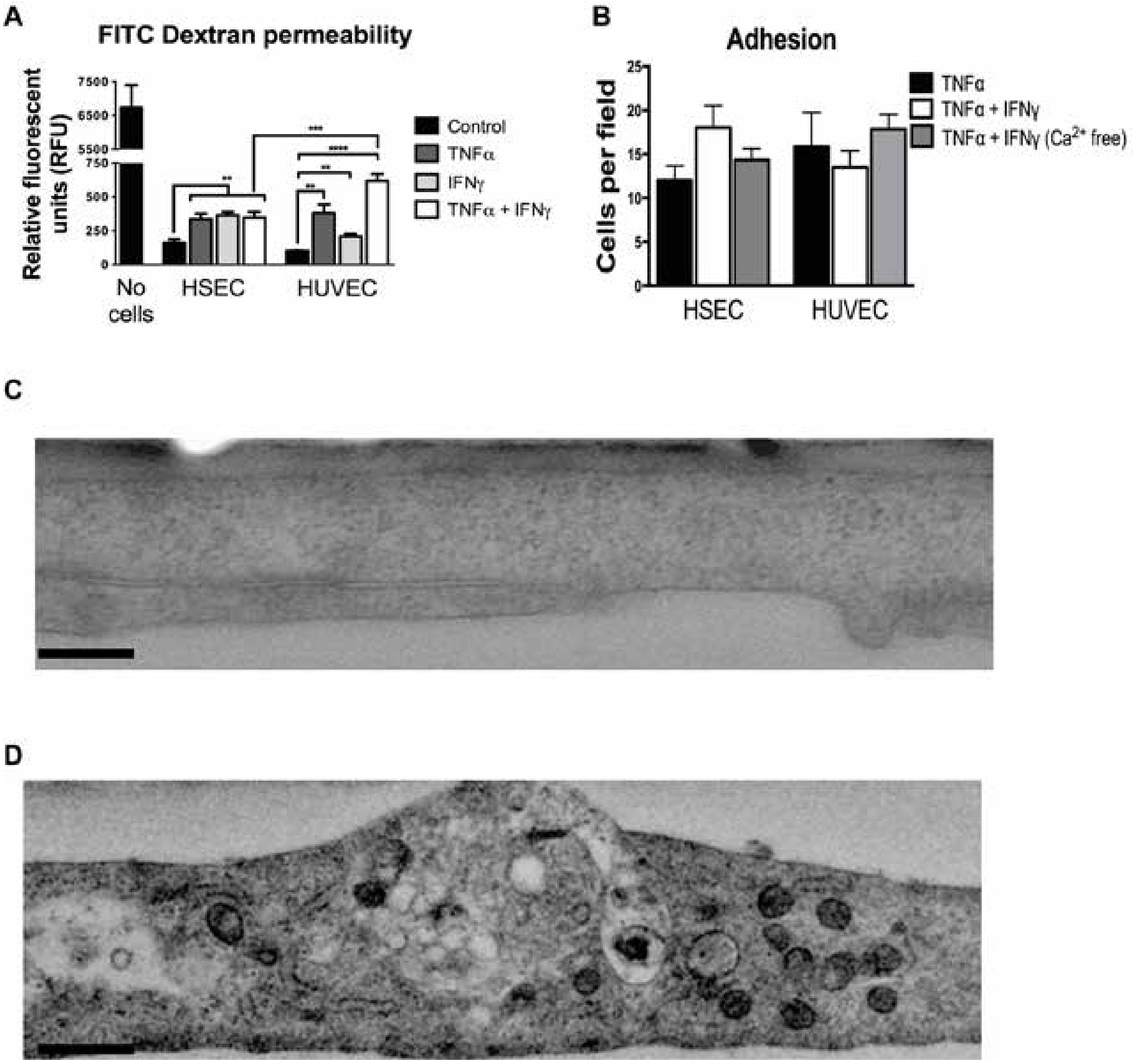

Permeability across monolayers of HSEC and HUVEC by a FITC-Dextran assay and adhesion of lymphocytes under shear stress. Ultrastructural changes in HSEC after cytokine stimulation. (A) Measurement of permeability by FITC Dextran assay across untreated or cytokine treated HSEC and HUVEC, results are the mean ± SEM of five independent experiments. (B) Quantification of adhesion of lymphocytes on cytokine treated HSEC and HUVEC monolayers cultured in flow media or calcium free flow media (Ca^{2+} free) with cytokine stimulation, results are the mean ± SEM of three independent experiments. Statistical significance was determined by 1-way ANOVA analysis, with a Tukey's *post-hoc* multiple comparison test . Representative transmission electron microscopy images of HSEC. (C) Cross sectional images of unstimulated HSEC monolayer and (D) Cross sectional images of HSEC monolayer pre-treated with TNFα and IFNγ for 24 hours. Bar 0.5 µm** P<0.005, *** P<0.001, ****P<0.0005.

Supplementary Figure 5

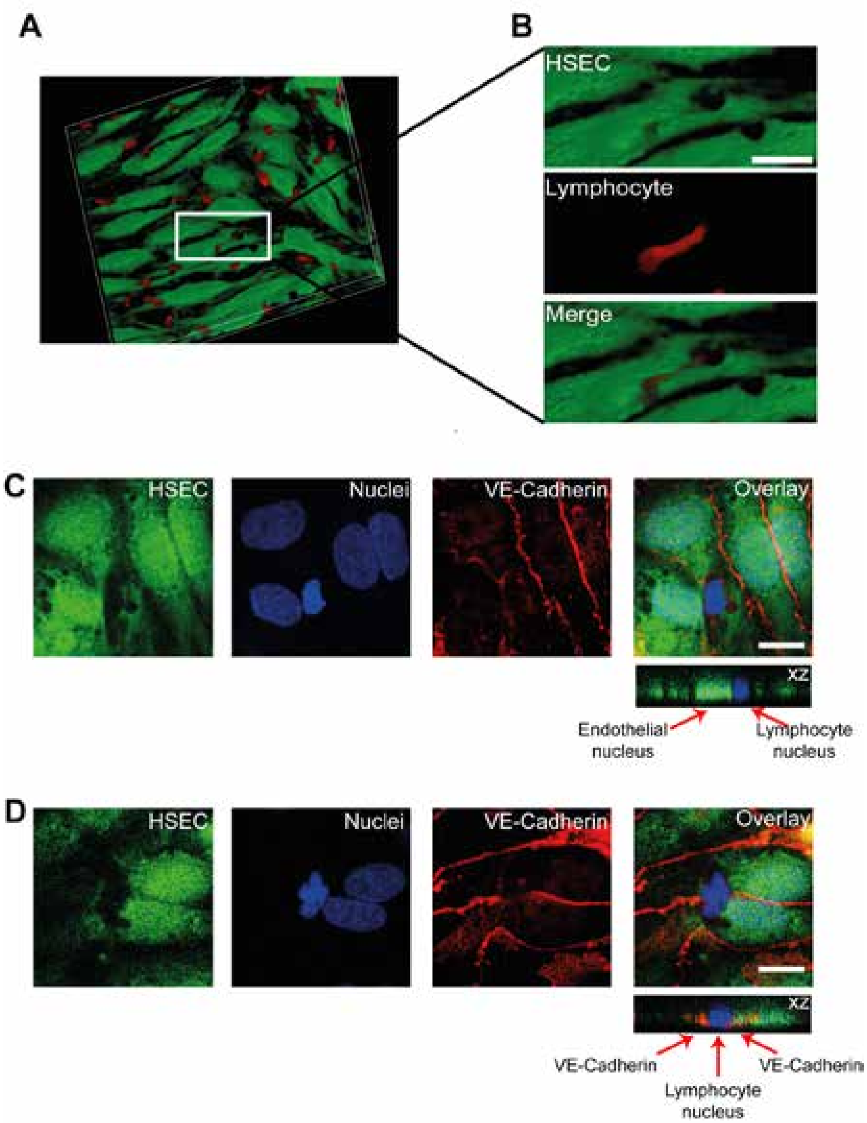

Intracellular crawling of lymphocytes across HSEC occurs in the presence of a chemokine gradient. (A) 3D reconstruction of an orthogonal (XZ) projection performed during live-cell imaging of peripheral blood lymphocytes migrating across TNFα and IFNγ stimulated HSEC undershear stress. HSEC were pre-labelled with CellTracker CFMDA (green) and lymphocytes with CellTracker BMQC (red). (B) Magnified images of boxed area separated into colours and merged image demonstrating a crawling lymphocyte (red) surrounded by endothelial cytoplasm (green). **(**C,D) Representative confocal images of lymphocytes adherent to TNFα and IFNγ treated HSEC monolayers grown on a collagen gel containing chemokine (IP-10). Endothelial cells were pre-labelled with CellTracker CFMDA (green), nuclei with DAPI (blue) and immunofluorescent staining with VE-Cadherin (red). Orthogonal (xz) projections are shown below each overlay image. Bar 10 µm (B,C,D).

Supplementary Figure 6

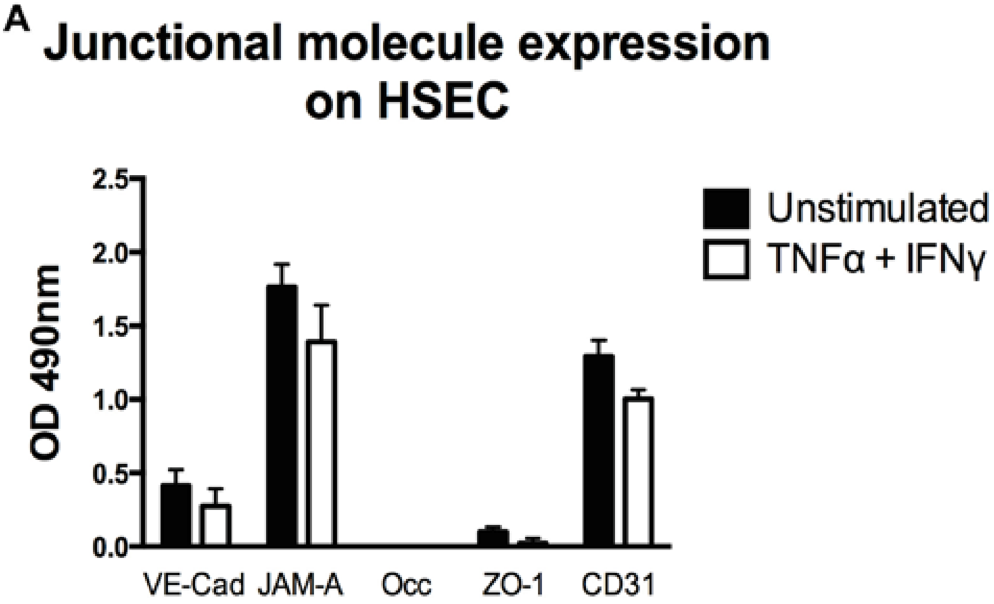

Junctional molecule expression in HSEC does not change significantly with TNFα and IFNγ stimulation. (A) Cell-based ELISA of junctional molecule expression in unstimulated and TNFα and IFNγ treated HSEC. Data are the mean of five experiments and values represent the mean optical density at 490nm of three replicate wells minus the optical density of an isotype matched control Ab. Statistical significance was determined by two-tailed Ttest.

Supplementary Table 1

Pathways upregulated in HSEC compared to HUVEC. Results of gene ontology analysis of the microarray data on genes which were significantly upregulated in HSEC compared to HUVEC.

GO Term	p-value
anatomical structure morphogenesis	6.81E-06
carboxylic acid metabolic process	1.00E-05
cell differentiation	5.52E-06
cellular developmental process	1.66E-05
cellular ketone metabolic process	1.05E-05
cellular lipid metabolic process	3.05E-07
developmental process	3.94E-06
enzyme regulator activity	1.00E-06
fatty acid metabolic process	1.30E-06
lipid metabolic process	1.04E-07
monocarboxylic acid metabolic process	8.63E-08
organ development	2.93E-06
organ morphogenesis	2.24E-06
oxoacid metabolic process	1.00E-05
plasma membrane part	1.14E-05
regulation of molecular function	1.64E-05
regulation of response to stimulus	1.13E-05
response to endogenous stimulus	2.15E-06
system development	8.35E-06

Supplementary Table 2

Pathways upregulated in HUVEC compared to HSEC. Results of gene ontology analysis of the microarray data on genes which were significantly upregulated in HUVEC compared to HSEC.

GO Term	p-value
acetylgalactosaminyltransferase activity	3.26E-05
adherens junction	1.09E-07
adherens junction organization	7.00E-05
aminoglycan biosynthetic process	3.15E-05
anatomical structure development	1.42E-15
anatomical structure formation involved in	1.68E-07

morphogenesis	
anatomical structure morphogenesis	1.86E-13
anchoring junction	1.57E-07
angiogenesis	4.02E-05
attachment of spindle microtubules to kinetochore	9.68E-05
axon guidance	2.95E-06
axonogenesis	4.08E-07
basolateral plasma membrane	9.12E-06
binding	7.56E-05
biological adhesion	5.59E-06
blood vessel development	2.47E-05
blood vessel morphogenesis	7.79E-05
cardiovascular system development	1.31E-07
cell adhesion	5.36E-06
cell adhesion molecule binding	2.79E-05
cell cycle	2.98E-13
cell cycle phase	3.28E-16
cell cycle process	1.27E-14
cell development	4.11E-09
cell differentiation	7.86E-10
cell division	1.07E-10
cell junction	4.33E-07
cell junction assembly	2.40E-06
cell junction organization	8.65E-07
cell leading edge	1.52E-05
cell migration	1.27E-05
cell morphogenesis	1.96E-07
cell morphogenesis involved in differentiation	6.04E-08
cell morphogenesis involved in neuron differentiation	2.93E-07
cell motility	7.05E-06
cell part morphogenesis	1.47E-06
cell projection	2.28E-06
cell projection morphogenesis	6.17E-07
cell projection organization	4.04E-07
cell projection part	1.32E-05
cell surface	4.42E-05
cell-cell adhesion	5.18E-05
cell-cell junction organization	6.52E-06
cell-substrate adherens junction	5.47E-06
cell-substrate junction	6.60E-06
cellular component assembly at cellular level	5.42E-05
cellular component morphogenesis	5.40E-07
cellular component movement	3.03E-06
cellular component organization	1.33E-10
cellular component organization at cellular level	1.81E-10

cellular component organization or biogenesis	9.41E-10
cellular component organization or biogenesis at cellular level	9.77E-10
cellular developmental process	1.61E-09
centrosome	4.93E-05
chondroitin sulfate proteoglycan metabolic process	1.29E-04
chordate embryonic development	3.39E-05
chromosome localization	2.22E-06
chromosome passenger complex	2.66E-05
chromosome segregation	3.38E-14
chromosome, centromeric region	1.28E-13
circulatory system development	1.31E-07
condensed chromosome	1.72E-13
condensed chromosome kinetochore	1.16E-14
condensed chromosome outer kinetochore	1.25E-08
condensed chromosome, centromeric region	1.30E-15
condensed nuclear chromosome	1.78E-05
condensed nuclear chromosome, centromeric region	2.74E-07
cytoplasm	1.31E-07
cytoplasmic part	2.03E-06
cytoskeletal part	8.20E-12
cytoskeletal protein binding	7.68E-06
cytoskeleton	1.93E-10
cytoskeleton organization	4.44E-09
developmental growth	8.34E-05
developmental process	8.89E-14
embryo development	9.37E-08
embryo development ending in birth or egg hatching	4.42E-05
embryonic morphogenesis	5.41E-06
establishment of chromosome localization	2.22E-06
extracellular matrix organization	2.72E-07
extracellular structure organization	6.49E-08
filopodium membrane	1.01E-04
focal adhesion	3.99E-06
generation of neurons	2.37E-08
glycoprotein biosynthetic process	4.45E-05
glycoprotein metabolic process	2.19E-05
glycosaminoglycan biosynthetic process	1.05E-04
intracellular non-membrane-bounded organelle	9.91E-06
kinetochore	8.75E-13
localization of cell	7.36E-06
locomotion	7.44E-07
M phase	5.17E-17
M phase of mitotic cell cycle	2.33E-16
metaphase plate congression	1.22E-05

microtubule	2.06E-06
microtubule binding	1.32E-04
microtubule cytoskeleton	5.55E-08
microtubule cytoskeleton organization	2.29E-09
microtubule motor activity	5.14E-05
microtubule organizing center	2.67E-06
microtubule-based process	2.04E-09
mitosis	1.80E-15
mitotic cell cycle	2.89E-16
mitotic metaphase plate congression	3.26E-05
mitotic sister chromatid segregation	1.33E-08
mitotic spindle organization	8.04E-09
multicellular organismal development	1.61E-13
multicellular organismal process	4.30E-07
Ndc80 complex	5.54E-06
negative regulation of cellular process	1.21E-04
negative regulation of locomotion	3.20E-05
negative regulation of protein phosphorylation	9.02E-05
nervous system development	4.15E-10
neurogenesis	1.63E-09
neuron development	1.14E-07
neuron differentiation	3.94E-07
neuron projection development	6.34E-07
neuron projection morphogenesis	1.41E-07
neuron recognition	8.75E-05
non-membrane-bounded organelle	9.91E-06
nuclear division	1.80E-15
organ development	5.50E-08
organelle fission	2.31E-16
peptidyl-proline dioxygenase activity	1.65E-05
peptidyl-proline hydroxylation	1.65E-05
plasma membrane part	4.44E-06
procollagen-proline dioxygenase activity	5.18E-06
protein binding	4.62E-07
protein localization to kinetochore	5.50E-05
proteoglycan biosynthetic process	4.70E-05
proteoglycan metabolic process	3.36E-06
regulation of actin filament bundle assembly	7.93E-05
regulation of cell cycle	5.51E-06
regulation of cell cycle process	6.02E-06
regulation of cell differentiation	4.32E-05
regulation of cell migration	7.96E-06
regulation of cell motility	2.91E-06
regulation of cellular component movement	1.01E-05
regulation of cellular component organization	1.45E-08

regulation of cytoskeleton organization	7.79E-06
regulation of developmental process	2.54E-05
regulation of locomotion	5.34E-06
regulation of MAPKKK cascade	3.17E-05
regulation of microtubule cytoskeleton organization	9.00E-06
regulation of microtubule-based process	8.36E-06
regulation of mitosis	1.89E-05
regulation of mitotic cell cycle	9.20E-05
regulation of multicellular organismal development	4.60E-06
regulation of multicellular organismal process	1.50E-06
regulation of nervous system development	5.07E-05
regulation of neurogenesis	1.12E-04
regulation of nuclear division	1.89E-05
regulation of organelle organization	8.52E-06
regulation of phosphate metabolic process	8.18E-05
regulation of phosphorus metabolic process	5.08E-05
regulation of phosphorylation	7.31E-05
regulation of protein phosphorylation	3.59E-05
regulation of stress fiber assembly	2.67E-05
sister chromatid segregation	4.05E-09
skeletal system development	3.42E-05
spindle	1.44E-08
spindle assembly	7.93E-05
spindle assembly involved in mitosis	4.34E-06
spindle checkpoint	1.56E-05
spindle microtubule	6.69E-05
spindle organization	2.74E-09
spindle pole	2.14E-08
sulfur compound metabolic process	1.52E-05
system development	5.49E-15
tissue development	8.14E-08
transferase activity, transferring glycosyl groups	2.74E-05
UDP-glycosyltransferase activity	3.39E-05
vasculature development	2.94E-06
wound healing	9.78E-06

Supplementary Table 3

Pathways exclusively downregulated in cytokine treated HUVEC. Results of gene ontology analysis of the microarray data on genes which were exclusively downregulated in TNFα and IFNγ challenged HUVEC compared to unstimulated HUVEC.

GO Term	p-value
adenyl nucleotide binding	8.41E-05
ATP catabolic process	9.65E-05
ATP-dependent chromatin remodeling	2.99E-11
attachment of spindle microtubules to chromosome	4.18E-05
attachment of spindle microtubules to kinetochore	2.21E-05
binding	7.48E-07
blood coagulation	2.34E-05
cell	9.21E-06
cell cycle	4.90E-44
cell cycle checkpoint	2.27E-05
cell cycle cytokinesis	4.93E-05
cell cycle phase	0
cell cycle process	5.47E-43
cell division	4.26E-25
cell part	9.21E-06
cellular component assembly	4.22E-09
cellular component assembly at cellular level	1.13E-10
cellular component biogenesis	9.86E-11
cellular component organization	8.01E-18
cellular component organization at cellular level	1.58E-20
cellular component organization or biogenesis	1.60E-19
cellular component organization or biogenesis at cellular level	1.34E-22
cellular macromolecular complex assembly	1.67E-13
cellular macromolecular complex subunit organization	3.56E-13
cellular macromolecule metabolic process	2.05E-08
cellular metabolic process	1.12E-06
cellular nitrogen compound metabolic process	1.32E-07
cellular process	6.03E-05
CenH3-containing nucleosome assembly at centromere	1.14E-11
centromere complex assembly	4.18E-05
centrosome	6.54E-15
centrosome cycle	1.51E-04
centrosome organization	2.68E-06
chromatin	1.46E-07
chromatin assembly	1.87E-13
chromatin assembly or disassembly	8.33E-13
chromatin modification	4.53E-05
chromatin organization	3.88E-07

chromatin remodeling	1.38E-07
chromatin remodeling at centromere	2.65E-12
chromatin silencing at rDNA	1.14E-05
chromosomal part	3.56E-28
chromosome	7.17E-30
chromosome condensation	9.17E-06
chromosome localization	2.39E-09
chromosome organization	2.79E-22
chromosome passenger complex	7.55E-05
chromosome segregation	2.10E-28
chromosome, centromeric region	2.26E-27
coagulation	2.34E-05
condensed chromosome	1.41E-31
condensed chromosome kinetochore	8.50E-29
condensed chromosome outer kinetochore	5.76E-11
condensed chromosome, centromeric region	2.51E-29
condensed nuclear chromosome	7.25E-09
condensed nuclear chromosome kinetochore	4.17E-07
condensed nuclear chromosome, centromeric region	5.53E-09
cytokinesis	6.50E-05
cytokinesis after mitosis	8.59E-06
cytoplasm	9.72E-10
cytoplasmic part	1.79E-07
cytoskeletal part	1.96E-11
cytoskeleton	4.26E-09
cytoskeleton organization	7.55E-09
cytosol	1.56E-09
DNA binding	1.50E-04
DNA conformation change	1.49E-16
DNA metabolic process	2.66E-18
DNA methylation on cytosine	3.94E-07
DNA packaging	7.43E-18
DNA recombination	2.00E-09
DNA repair	2.43E-09
DNA replication	1.57E-10
DNA replication-dependent nucleosome assembly	1.76E-07
DNA replication-dependent nucleosome organization	1.76E-07
DNA replication-independent nucleosome assembly	1.04E-11
DNA replication-independent nucleosome organization	1.04E-11
DNA strand elongation	8.49E-06
DNA strand elongation involved in DNA replication	4.55E-06
DNA-dependent DNA replication	1.59E-06
embryo development	2.59E-05
establishment of chromosome localization	2.39E-09
establishment of mitotic spindle localization	1.82E-07

establishment of mitotic spindle orientation	1.00E-05
establishment of organelle localization	3.36E-09
establishment of spindle localization	4.50E-06
establishment of spindle orientation	3.58E-05
G1/S transition of mitotic cell cycle	8.15E-08
G2/M transition of mitotic cell cycle	4.96E-12
hemostasis	3.09E-05
histone exchange	6.71E-12
interphase	6.73E-17
interphase of mitotic cell cycle	4.82E-17
intracellular	6.81E-17
intracellular membrane-bounded organelle	4.47E-16
intracellular non-membrane-bounded organelle	2.49E-26
intracellular organelle	7.31E-21
intracellular organelle lumen	6.30E-19
intracellular organelle part	9.42E-20
intracellular part	8.91E-18
kinesin complex	1.20E-05
kinetochore	1.29E-25
kinetochore organization	2.37E-06
M phase	4.06E-39
M phase of meiotic cell cycle	1.41E-08
M phase of mitotic cell cycle	7.57E-36
macromolecular complex	9.06E-09
macromolecular complex assembly	1.69E-09
macromolecular complex subunit organization	3.47E-10
macromolecule metabolic process	9.59E-07
meiosis	1.41E-08
meiosis I	8.12E-05
meiotic cell cycle	1.49E-09
meiotic chromosome segregation	1.02E-04
membrane-bounded organelle	8.49E-14
membrane-enclosed lumen	3.38E-19
metabolic process	5.39E-06
metaphase plate congression	5.59E-09
microtubule	9.25E-06
microtubule anchoring	4.93E-05
microtubule binding	1.63E-05
microtubule cytoskeleton	2.11E-17
microtubule cytoskeleton organization	9.68E-19
microtubule organizing center	7.38E-16
microtubule organizing center organization	1.99E-06
microtubule polymerization or depolymerization	1.02E-04
microtubule-based process	1.07E-14
midbody	1.13E-06

mitosis	5.88E-35
mitotic cell cycle	0
mitotic cell cycle spindle assembly checkpoint	1.16E-04
mitotic chromosome condensation	7.35E-05
mitotic metaphase plate congression	9.94E-09
mitotic recombination	6.25E-06
mitotic sister chromatid segregation	3.09E-19
mitotic spindle organization	3.73E-08
ncRNA metabolic process	6.71E-05
Ndc80 complex	1.59E-05
negative regulation of cell cycle process	7.18E-06
negative regulation of cell division	7.35E-05
negative regulation of gene expression, epigenetic	1.09E-04
negative regulation of mitosis	4.26E-05
negative regulation of mitotic metaphase/anaphase transition	3.17E-05
negative regulation of nuclear division	4.26E-05
nitrogen compound metabolic process	2.71E-07
non-membrane-bounded organelle	2.49E-26
nuclear chromosome	5.82E-13
nuclear chromosome part	1.20E-07
nuclear division	5.88E-35
nuclear lumen	3.02E-22
nuclear part	1.44E-23
nucleic acid metabolic process	1.87E-08
nucleobase, nucleoside, nucleotide and nucleic acid metabolic process	6.16E-08
nucleolar part	1.11E-04
nucleolus	1.36E-07
nucleoplasm	6.49E-17
nucleosome	2.11E-08
nucleosome assembly	8.48E-14
nucleosome organization	3.66E-12
nucleus	1.83E-21
organelle	6.94E-19
organelle assembly	2.34E-05
organelle fission	2.85E-34
organelle localization	2.15E-08
organelle lumen	1.10E-18
organelle organization	7.05E-24
organelle part	9.54E-20
primary metabolic process	3.13E-06
protein binding	2.78E-12
protein complex	8.62E-07
protein localization to chromosome	1.28E-05
protein localization to kinetochore	4.02E-07

protein-DNA complex	1.49E-08
protein-DNA complex assembly	2.16E-15
protein-DNA complex subunit organization	7.90E-14
regulation of attachment of spindle microtubules to kinetochore	1.58E-06
regulation of body fluid levels	7.80E-05
regulation of cell cycle	4.21E-10
regulation of cell cycle arrest	2.36E-05
regulation of cell cycle process	6.56E-11
regulation of chromosome segregation	2.10E-09
regulation of cytokinesis	1.34E-04
regulation of microtubule cytoskeleton organization	7.40E-05
regulation of microtubule-based process	3.13E-05
regulation of mitosis	1.27E-09
regulation of mitotic cell cycle	3.56E-09
regulation of mitotic metaphase/anaphase transition	3.79E-08
regulation of nuclear division	1.27E-09
regulation of transcription involved in G1/S phase of mitotic cell cycle	2.05E-06
replication fork	1.10E-05
response to DNA damage stimulus	1.03E-06
sister chromatid segregation	1.17E-19
spindle	3.59E-19
spindle assembly	1.67E-06
spindle assembly checkpoint	2.37E-05
spindle assembly involved in mitosis	3.60E-05
spindle checkpoint	5.81E-07
spindle localization	1.28E-05
spindle microtubule	1.31E-06
spindle midzone	6.15E-06
spindle organization	1.06E-11
spindle pole	2.80E-13
structure-specific DNA binding	1.19E-05
telomere maintenance	3.28E-06
telomere maintenance via recombination	3.60E-05
telomere maintenance via semi-conservative replication	5.68E-06
telomere maintenance via telomere lengthening	1.14E-05
telomere organization	8.55E-07
wound healing	1.26E-05

Supplementary Table 4

Probe mixes for qRT-PCR used in this study.

	**ThermoFisher Scientific**
Target	Taqman Assay ID
CXCL9	Hs00171065_m1
CXCL10	Hs01124252_g1
CXCL11	Hs04187682_g1
ICAM-1	Hs00164932_m1
Stabilin-1	Hs01109068_m1
Occludin	Hs00170162_m1
CD36	Hs00169627_m1
Mannose Receptor	Hs00267207_m1
Podoplanin	Hs00366766_m1

Supplementary Material and Methods

Isolation of peripheral blood lymphocytes (PBL)

PBLs were isolated as previously described (1) by density gradient centrifugation over Lympholyte (VH Bio, Gateshead, U.K.) for 25 min at 800g. Harvested lymphocytes were washed and resuspended in RPMI1640/10% FCS, monocytes were depleted by plastic adherence.

Antibodies and Immunostaining

A mAb against Stabilin-1 (9-11) has been described (2) and was used at 10µg/ml. The lysosomal compartment was identified by antibodies against VAMP-7 (OSS00047G, 10 µg/ml, ThermoFisher Scientific, Cramlington, UK) and CD63 (NK1/C3, 5 µg/ml Abcam, Cambridge, United Kingdom). Antibodies against junctional molecules included VE-Cadherin (ab33168, 5 µg/ml), Occludin (ab31721, 5 µg/ml) both from Abcam). JAM-A (361700, 5 µg/ml) ZO-1 (1A12, 5 µg/ml), E-Cadherin (Sec11, 5 µg/ml), Claudin-1 (2H10D10, 5 µg/ml) all from Life Technologies, Paisley UK; CD31 (JC70A, 2.5 µg/ml) Dako, Ely, UK). Antibodies against lymphocyte marker CD3 (UCHT-1, 5 µg/ml eBiosciences Hatfield United Kingdom) and Kupffer cell marker CD68 (Y1/82A, 5 µg/ml, BD Biosciences). Control antibodies were Rat IgG2a (eBioscience), mouse IgG1 (Dako), mouse IgG2b (R and D systems), Rabbit polyclonal (Dako). Alexa Fluor 488-conjugated goat anti-rat IgG, Alexa Fluor 546- conjugated goat anti-mouse IgG1, Alexa-Fluor 546-conjugated goat anti-rabbit IgG and Alexa-Fluor 633 goat anti-mouse Ig2b were all from Thermo Fisher.

Fluorescent labeling

For immunofluorescent staining, acetone-fixed cryosections were initially incubated with 10% normal goat serum in PBS buffer for 30 min before the addition of primary mouse anti-human mAbs in PBS for 60 min at room temperature in a humidified container. After washing in PBS, the sections were incubated with the relevant fluorescent conjugated goat anti-mouse, rat or rabbit secondary for 30 min in PBS. Sections were washed in PBS and mounted with fluorescence mounting medium (Dako). For staining of HSEC, cells were seeded at confluence on rat tail collagen- coated chamberslides (μ-slide 8 well Ibidi , Thistle Scientific, Glasgow UK). and incubated overnight. The cells were fixed with 4% paraformaldehyde solution in PBS and permeabilized with 0.3% Triton X-100. After incubation with 10% normal goat serum in PBS buffer for 30 min, cells were incubated with primary mAb or isotype-matched control for 1 h at room temperature. Cells were then incubated with fluorescent secondary Abs for 30 minutes before being mounted with fluorescence mounting medium (Dako). Cells were imaged by confocal microscopy on a Zeiss 780 Zen microscope.

Measurement of junctional molecule expression on HSEC and HUVEC by ELISA

Endothelial cells were grown to confluence in collagen-coated 96-well flat-bottom plates and fixed with methanol before performing ELISA. Cells were left under basal conditions or stimulated with cytokines (10 ng/ml recombinant human TNF-α, 10 ng/ml recombinant human IFN-γ). All stimulations were for 24 h. Cells were preincubated with 2% goat serum (Sigma-Aldrich) for 1 h.

This was followed by incubation with anti-human primary Ab VE-Cadherin (ab33168, 5 μg/ml), Occludin (ab31721, 5 μg/ml) both from Abcam, Cambridge UK. JAM-A (361700, 5 μg/ml), ZO-1 (1A12, 5 μg/ml) both from Life Technologies, Paisley UK, CD31 (JC70A, 2.5 μg/ml) Dako, Ely, UK) and control Ab (mouse IgG1, 10 μg/ml; Dako, Rabbit polyclonal 5 μg/ml; Dako) for 45 min at room temperature. The cells were then washed and incubated with a peroxidase-conjugated goat anti-mouse or goat anti-rabbit secondary Ab (P0447 or P0449 1/500; Dako) for 45 min at room temperature. The ELISA was developed using *O*-phenylenediamine substrate (Dako) according to the manufacturer's instructions. Junctional molecule expression was expressed as the mean absorbance from three replicate wells minus the absorbance of an isotype-matched control Ab.

Flow-based adhesion assay.

In other experiments HSEC were stimulated for 24 hours with TNF-α (10ng/ml) and the addition of IFNα (10ng/ml), IFNβ (10ng/ml) IFNω, IFN λ1, IFNλ2 (all 10ng/ml);Peprotech or Lipopolysaccharide (1mg/ml); Sigma. For experiments with calcium-free media endothelial monolayers were cultured in minimal essential medium eagle spinner modification (Sigma), containing 1% non essential amino acids, 5% FCS and 2mM L glutamine, 0.5mM EGTA. Lymphocytes (1×10^6 cells/ml) were perfused through the microslide over the endothelial cells at a shear stress of 0.05 Pa.

HSEC were prelabeled with CellTracker Green CMFDA (Thermo Fisher) per the manufacturer's instructions followed by flow assays with lymphocytes, as described above, for 10 min. Cells were then fixed in 4% paraformaldehyde and permeabilized with 0.3% Triton X-100. Cells were stained with a DAPI

stain (Thermo Fisher) for nuclear staining. Slides were then examined using a Zeiss 780 ZEN microscope. Confocal images and z-stacks were acquired and analyzed by ZEN software. We counted the number of lymphocytes undergoing intracellular migration in randomly selected high-power fields and the total number of lymphocytes adherent in each field. The rate of intracellular migration was then expressed as a proportion of the total number of lymphocytes counted. HSEC monolayers were incubated with blocking Abs against CLEVER-1/stabilin-1 (3-372, 20 µg/ml), ICAM-1 (10µg/ml;R and D systems), PD-L1 (10F.9G2, 10µg/ml Biolegend, London UK) or isotype-matched control (mouse IgG1, Dako and mouse IgG2b R and D systems). Alternatively HSEC were pre-treated with Blebbistatin (50 µM for 15 minutes) or Cytochalasin D (5 µM for 2 minutes). In other experiments lymphocytes were incubated with blocking antibody against CXCR3 (49801, 10µg/ml R and D systems) or isotype-matched control (mouse IgG1,Dako).

HSEC in flow chambers were then stained with following antibodies: polyclonal Ab for VE-Cadherin (ab33168, 5 µg/ml, Abcam), CD31 (JC70A, 2.5 µg/ml) Dako), alpha tubulin (ab7291, 1 µg/ml, Abcam) or CD4 (OKT4, 5 µg/ml) eBioscience ; followed by Alexa Fluor 546-conjugated goat anti-rabbit or Alexa Fluor 546-conjugated goat anti-mouse IgG1 (Thermo Fisher). In other experiments chambers were stained for actine with Alex Fluor 633 phalloidin (Thermo Fisher). Cells were stained with a DAPI stain (Thermo Fisher) for nuclear staining.

Quantification of Actin and Microtubule fibres in HSEC.

Images were taken of monolayers of HSEC labeled for actin or microtubules

as outlined above. Zstack images were taken of random fields. For each field 10 orthogonal projections were saved and threshold analysis performed with ImageJ software version 1.42q (NIH).

Collagen chamberslide assay

Collagen gels (3mg/ml) were set up in chamberslides, containing chemokine CXCL10 (10ng/ml, Peprotech, UK) and then layered with fibronectin (50µg/ml). HSEC monolayer was grown to confluence over the gels. HSEC monolayers were pretreated with TNF-α and IFN-γ for 24 hours and prelabeled with CellTracker Green CMFDA (Thermo Fisher). Peripheral blood lymphocytes (1 × 10^6 cells/ml) were overlaid on HSEC for 20 minutes and then non-adherent lymphocytes were washed with PBS and cells were then fixed in 4% paraformaldehyde. HSEC were then stained with polyclonalAb for VE-Cadherin (ab33168, 5 µg/ml, Abcam) followed by Alexa Fluor 546-conjugated goat anti-rabbit. Cells were stained with a DAPI stain (Thermo Fisher) for nuclear staining.

Electron Microscopy

HSEC were seeded at confluence on at confluence on rat tail collagen-coated coverslips and then cytokine-stimulated for 24 hours. Following this peripheral blood lymphocytes (1 × 10^6 cells/ml) were overlaid on HSEC for 20 minutes and then non-adherent lymphocytes were washed with PBS. The cells were then fixed at room temperature in 2.5% EM grade gluteraldehyde buffer followed by secondary fixation in osmium tetroxide. The samples were then dehydrated and embedded. Sections were cut using a diamond knife and stained with uranyl acetate and lead citrate. Sections were visualized with a

JEOL electron microscope.

Quantitative real-time PCR

Total RNA was isolated and purified from cell pellets of HSEC and HUVEC using the RNeasy® Mini Kit (Qiagen) and RNase-Free DNase Set (Qiagen), following the manufacturer's protocol. RNA quantity and purity was determined with a Nanophotometer™ (Implen GmbH) and cDNA was synthesised using SuperScript®II Reverse Transcriptase (Thermo Fisher). mRNA expression of target proteins was assessed by quantitative real-time PCR (qPCR), utilising predesigned TaqMan® Gene Expression Assays (Applied Biosystems®) (as detailed in supplemental Table 4) and 2X TaqMan® Universal PCR Master Mix (Applied Biosystems®). qPCR was performed on a Roche Lightcycler 480 (Roche) using the following programme: 95°C for 10 min and 45 cycles of 95 °C for 10 s, 60 °C for 1 min, 72 °C for 1 s. Target mRNA levels were normalised to the housekeeping gene (GAPDH) and a fold change of relative expression from the appropriate unstimulated control was calculated utilising the $2^{-\Delta\Delta Ct}$ method (3).

Permeability assays

Rat tail collagen (1 in 100; Sigma-Aldrich)-coated 24-well Millicell transwell inserts (polyethylene terephthalate, 0.4 µm; EMD Millipore) were seeded with 1.5 x 10^5 HSEC or HUVEC cells and cultured overnight to confluence. Subsequently, cell monolayers were subjected to cytokine stimulation or relevant media control and incubated for a further 24 h. Transendothelial electrical resistance (TEER) was measured using a Millicell-ERS2 Volt-Ohm

Meter (EMD Millipore) and expressed in Ωcm^2. Alternatively, 1 mg/ml FITC-dextran (Sigma-Aldrich) was added to the apical surface of the transwell insert and incubated at 37 °C for 1 h. Following this, the fluorescence of the basolateral media was measured (excitation λ = 485 nm, emission λ = 520 nm) and was expressed in relative fluorescent units (RFU).

Microarray

For microarray analysis, total RNA was isolated as described above from HUVEC and HSEC batches which were untreated and batches which were challenged with TNF-α and IFN-γ for 24 h at 10 ng/ml, two replicates were used for each condition. Microarray fabrication, hybridization and detection were conducted at the Agilent Microarray service, University of Birmingham. UK. Briefly, the Low-input Quick Amp Labeling Kit, One-Color generated fluorescent RNA (complimentary RNA). The method utilized a T7 RNA polymerase blend, which simultaneously amplified target material and incorporated Cyanine 3-CTP. The microarrays were washed and scanned Data were extracted through GeneSpring software, Agilent Technologies. Fold change >2 with adjusted $P<0.05$ was considered for gene changes. Gene ontology analysis was also performed with the GeneSpring software on genes that were significantly differently expressed.

Microarray data submission to public repository

As per recommendations made by the Microarray Gene Expression Data society, our microarray data has been deposited with the Gene Expression Omnibus, accession number GSE78020.

1. Curbishley SM, Eksteen B, Gladue RP, Lalor P, Adams DH. CXCR 3 activation promotes lymphocyte transendothelial migration across human hepatic endothelium under fluid flow. Am J Pathol 2005;167:887-899.
2. Karikoski M, Marttila-Ichihara F, Elima K, Rantakari P, Hollmen M, Kelkka T, Gerke H, et al. Clever-1/stabilin-1 controls cancer growth and metastasis. Clin Cancer Res 2014;20:6452-6464.
3. Livak KJ, Schmittgen TD. Analysis of relative gene expression data using real-time quantitative PCR and the 2(-Delta Delta C(T)) Method. Methods 2001;25:402-408.
